# Cadherin-2 Controls Directional Chain Migration of Cerebellar Granule Neurons

**DOI:** 10.1371/journal.pbio.1000240

**Published:** 2009-11-10

**Authors:** Sandra Rieger, Niklas Senghaas, Axel Walch, Reinhard W. Köster

**Affiliations:** 1 Institute of Developmental Genetics, Helmholtz Zentrum München, German Research Center for Environmental Health, Munich-Neuherberg, Germany; 2 Institute of Pathology, Helmholtz Zentrum München, German Research Center for Environmental Health, Munich-Neuherberg, Germany; Cambridge University, United Kingdom

## Abstract

Long distance migration of differentiating granule cells from the cerebellar upper rhombic lip has been reported in many vertebrates. However, the knowledge about the subcellular dynamics and molecular mechanisms regulating directional neuronal migration in vivo is just beginning to emerge. Here we show by time-lapse imaging in live zebrafish (*Danio rerio*) embryos that cerebellar granule cells migrate in chain-like structures in a homotypic glia-independent manner. Temporal rescue of zebrafish Cadherin-2 mutants reveals a direct role for this adhesion molecule in mediating chain formation and coherent migratory behavior of granule cells. In addition, Cadherin-2 maintains the orientation of cell polarization in direction of migration, whereas in Cadherin-2 mutant granule cells the site of leading edge formation and centrosome positioning is randomized. Thus, the lack of adhesion leads to impaired directional migration with a mispositioning of Cadherin-2 deficient granule cells as a consequence. Furthermore, these cells fail to differentiate properly into mature granule neurons. In vivo imaging of Cadherin-2 localization revealed the dynamics of this adhesion molecule during cell locomotion. Cadherin-2 concentrates transiently at the front of granule cells during the initiation of individual migratory steps by intramembraneous transport. The presence of Cadherin-2 in the leading edge corresponds to the observed centrosome orientation in direction of migration. Our results indicate that Cadherin-2 plays a key role during zebrafish granule cell migration by continuously coordinating cell-cell contacts and cell polarity through the remodeling of adherens junctions. As Cadherin-containing adherens junctions have been shown to be connected via microtubule fibers with the centrosome, our results offer an explanation for the mechanism of leading edge and centrosome positioning during nucleokinetic migration of many vertebrate neuronal populations.

## Abstract 2

Imaging cerebellar granule neurons in zebrafish embryos reveals a further role for Cadherin-2 in neurogenesis: regulating cohesive and directional granule cell migration via intra-membranous Cadherin-2 relocalisation and centrosome stabilization.

## Author Summary

As the vertebrate nervous system develops, neurons migrate from proliferation zones to their later place of function. Adhesion molecules have been implicated as key players in regulating cellular motility. In addition, the centrosome (the main microtubule organizing center of the cell) orients into the direction of neuronal migration. In this study we assign the trans-membrane adhesion molecule Cadherin-2 with an important function in the migration of granule neurons in the cerebellum, by interconnecting adhesion with directionality of migration. Time-lapse analysis in transparent zebrafish embryos revealed that Cadherin-2 enables granule neurons to form ‘chain’-like structures during migration. In addition, this adhesion molecule stabilized the position of the centrosome at the leading edge of the migrating neuron. In vivo tracing of a fluorescent Cadherin-2 reporter molecule showed that during individual migratory steps of a granule neuron, Cadherin-2 is shifted along the cell membrane in contact with chain-migrating neighboring neurons to the front compartment of migrating cells. Cadherin-2 is a crucial component of adherens junctions, which are connected via microtubules to the centrosome. We propose that the forward translocation of Cadherin-2-containing adherens junctions stabilizes the centrosome to the cell's front. Cadherin-2 thus transmits cell-cell contact modulation into directional migration of cerebellar granule neurons.

## Introduction

The adhesion molecule family of classic cadherins is known to play important roles during many steps of central nervous system (CNS) development. For example in vertebrates they are critically involved in the formation of the neural tube, morphogenesis and maintenance of brain compartments, the regulation of neuronal migration, the elongation and fasciculation of axons, as well as the establishment of dendritic spines and synapses [1]–[5].

Cadherin-2 (N-Cadherin) is the most common classic Cadherin in the vertebrate CNS, being expressed broadly throughout the developing and mature brain among other tissues. Inactivation of Cadherin-2 in mice and zebrafish results in severe neural tube formation defects [6],[7]. This lack of structural integrity makes it difficult to assess the specific functions of Cadherin-2 during later stages of brain differentiation. Conditional inactivation of Cadherin-2 in the CNS via application of function-interfering antibodies or dominant-negative variants have revealed crucial roles for this adhesion molecule in neural crest delamination [1],[8]. More recently, disruption of Cadherin-2 function specifically in migrating chick neural crest cells reduced their migratory velocity [9]. Similarly, precerebellar neurons in the caudal hindbrain migrate at reduced speed, when Cadherin-2 function is impaired [3]. Thus, whereas classical studies have attributed Cadherin-2 function mostly to rigid cell-cell adhesion between stationary cells in mediating tissue integrity and segregation of different cell populations [10], the importance of Cadherin-2 in regulating cellular motility and in particular neuronal migration is emerging.

Cerebellar granule cells (GCs) are an excellent model to investigate the cellular and molecular mechanisms underlying neuronal migration. Quantitative Cadherin expression studies by flow cytometry suggested a correlation of Cadherin-2 expression with GC migration in the mouse cerebellum [11]. Moreover, Cadherin-2 antagonizing peptides affected the orientation of GCs in cerebellar slice cultures arguing for a direct role of Cadherin-2 in regulating the directional migration of GCs [12]. The underlying cellular mechanisms through which Cadherin-2 could affect migration remain, however, elusive.

The single transmembrane classic Cadherins, like Cadherin-2, are adherens junction core components and mediate heterotypic and homotypic cell adhesion via the formation of Cadherin cis- and trans-dimers [13]. Intracellularly, the cytoplasmic domain of Cadherins interacts via a- and b-catenins with the actin cytoskeleton [14]. Besides their attachment to neighboring cells, adherens junctions have been implicated in regulating cell polarity, as their loss results in poorly polarized cellular morphologies and failures in polarized cell behavior [15],[16]. Intriguingly, Cadherin-2 indirectly interacts with the microtubule cytoskeleton through the scaffolding protein IQGAP1, which has been recently shown to influence cerebellar GC migration [17],[18]. Many tangentially and radially migrating neurons including cerebellar GCs migrate via nucleokinesis, marked by the characteristic forward transport of the nucleus along microtubule fibers. A key component of this migratory mode during nucleokinetic migration is the centrosome, or microtubule organizing center (MTOC), which positions in front of the nucleus to initiate and orchestrate directional nucleokinetic migration [19]–[22]. Not much is known about how the centrosome remains in front of the nucleus to regulate directionality during cerebellar GC migration. Given the potential of Cadherin-2 to regulate GC migration and orientation [12], this molecule is a good candidate to influence centrosomal positioning and thus directionality of migrating GCs.

Disruption of Cadherin-2 function in zebrafish embryos results in severe cerebellar defects [7],[23]. While these gross malformations are likely caused by neurulation defects, the mispositioning of neurons throughout the brain in homozygous Cadherin-2 mutants, *parachute* (*pac*), suggests a role for zebrafish Cadherin-2 in regulating neuronal migration. We have recently shown that GFP-expression in the cerebellum of the zebrafish transgenic line gata1:GFP, due to a random transgene integration is confined to all migrating and differentiating GCs, and we have characterized their migration pathways in detail [24],[25]. Building on these in vivo time-lapse imaging results, we now show that Cadherin-2 regulates the coherence and directionality of GC migration and we offer an explanation of how Cadherin-2-mediated adhesion may contribute to centrosome positioning during homophilic GC migration in zebrafish.

## Results

### Cerebellar GCs Migrate in a Homophilic Manner

**Figure 1:**
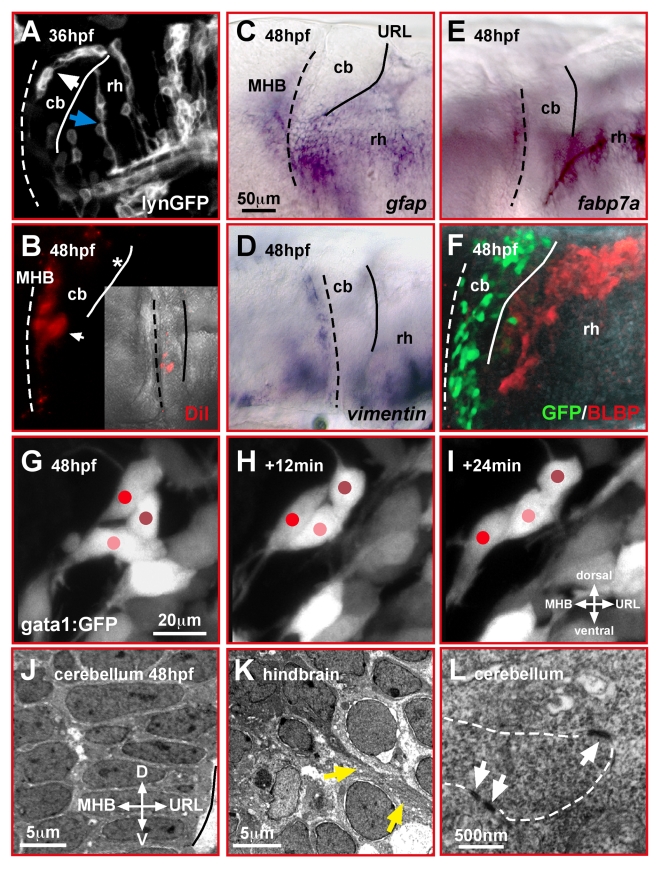
Migrating cerebellar GCs interact in a homophilic manner. (A–F) Lateral view of the zebrafish cerebellum and caudal hindbrain. Neither mosaic expression of membrane-targeted lynGFP (A) nor DiI-injection into the URL (B) reveals glia-like cell morphologies in the cerebellum, unlike that found in the hindbrain (blue arrow). In the cerebellum, GCs emigrating from the URL form chain-like structures (white arrow). Expression of the glia markers *gfap* (C), *vimentin* (D), *fabp7a* (E), and BLBP-immunohistochemistry in gata1:GFP embryos (F) supports that glia cells are absent in the cerebellum during GC migration. (G–I) In vivo time-lapse images (lateral view) of fluorescent GCs in gata1:GFP WT embryos reveals intense interactions among migrating GCs, which alternate in forward movements and resting behavior (see Video S1). (J–L) The differentiating cerebellum shows a highly ordered cellular arrangement when analyzed by TEM (J), with direct contacts of cerebellar cells via adherens junctions (L, white arrows). In the hindbrain (K), in contrast, fiber-like processes (yellow arrows) are present between individual cells (see also A, blue arrow). The MHB and URL are marked with a dashed and solid line, respectively. cb, cerebellum; MHB, midbrain-hindbrain boundary; rh, rhombencephalon.

To analyze the migration behavior of immature cerebellar GCs in zebrafish embryos, we utilized the stable gata1:GFP transgenic line (strain 781). In this line, GFP is expressed in cerebellar GCs due to a position effect apart from its inherent expression in developing erythrocytes. In a previous characterization we showed that strong GFP expression initiates in migrating GCs shortly before GCs become post-mitotic and start to differentiate [25]. We initially observed that GCs migrate in chain-like structures toward the midbrain-hindbrain boundary (MHB). To investigate these cellular interactions in more detail, we first addressed whether glial cells are present in the zebrafish cerebellum and hindbrain before and during onset of GC migration (36 hpf, 48 hpf). To mosaically label individual cells, we injected mRNA encoding for membrane-targeted lynGFP [26] into one blastomere of 8-cell stage zebrafish embryos. In vivo confocal microscopy revealed that in rhombomeres posterior to the cerebellum, cells reminiscent of radial glia were attached to the apical and basal membrane via long processes with typical endfeet-like structures. In some cases, individual cells appeared to move along these processes (Figure 1A, blue arrow). In contrast, such cell morphologies could not be observed in the developing cerebellum. Rather, cells formed chain-like structures (Figure 1A, white arrow), when emanating from the cerebellar rhombic lip (also termed upper rhombic lip, URL). These cells migrated toward and along the MHB (Figure 1B, white arrow) as shown previously [27]. Similarly, DiI-injections at 48 hpf into the URL (Figure 1B, white asterisk) never revealed any cells with glial-like morphologies. This suggests that migrating URL-derived cells in the differentiating zebrafish cerebellum migrate in the absence of a prominent migration-supporting glial fiber meshwork.

To substantiate our findings, we examined the expression of the glial cell-specific genes *gfap*, *vimentin*, and *fabp7a* at the onset of GC migration (48 hpf). Expression of these glial markers was prominent in the rhombencephalon but absent in the differentiating cerebellum (Figure 1C–1E) persisting up to 100 hpf (unpublished data), when GC migration has largely ceased [25]. Furthermore, immunohistochemistry against Brain Lipid-Binding Protein (BLBP), which is activated in glia during stages of neuronal migration [28], indicated that glial cells are largely absent from the differentiating cerebellum of gata1:GFP transgenic embryos and are not associated with the chain-like structures of migratory GCs (Figure 1F). In addition, microangiography using intracardial quantum dot injections showed that during developmental stages with prominent GC migration only the dorsal longitudinal vein (DLV) between both cerebellar halves is present, therefore excluding blood vessels as migration guiding meshwork for embryonic GCs (unpublished data, see also Movie 6 in [29]).

To directly reveal the migratory behavior of zebrafish GCs, we performed intravital time-lapse confocal microscopy at high magnification in gata1:GFP transgenic embryos. These studies demonstrated that GCs migrate from the URL toward the MHB (Figure 1G–1I, see Video S1, *n* = 6), by gliding along one another and forming dynamic chain-like structures. These findings support that zebrafish cerebellar GCs migrate by homophilic interactions.

Intriguingly, transmission electron microscopy (TEM) at 48 hpf revealed a highly oriented organization of cerebellar cells from the URL to the MHB (Figure 1J). While in the rhombencephalon fiber-like processes were observed between hindbrain cells (Figure 1K, yellow arrows), dorsal cerebellar migratory neurons displayed many direct adherens junction-mediated cell-cell contacts (Figure 1L, white arrows). This confirms the predominance of homophilic interactions between GCs. In addition, these findings suggest that cohesion—the ability of cells to aggregate into distinct clusters [30]—is likely important for the coordinated movement of zebrafish GCs and involves the regulation of their adhesive properties.

### Cadherin-2 Is a Likely Candidate to Mediate GC Migration

**Figure 2:**
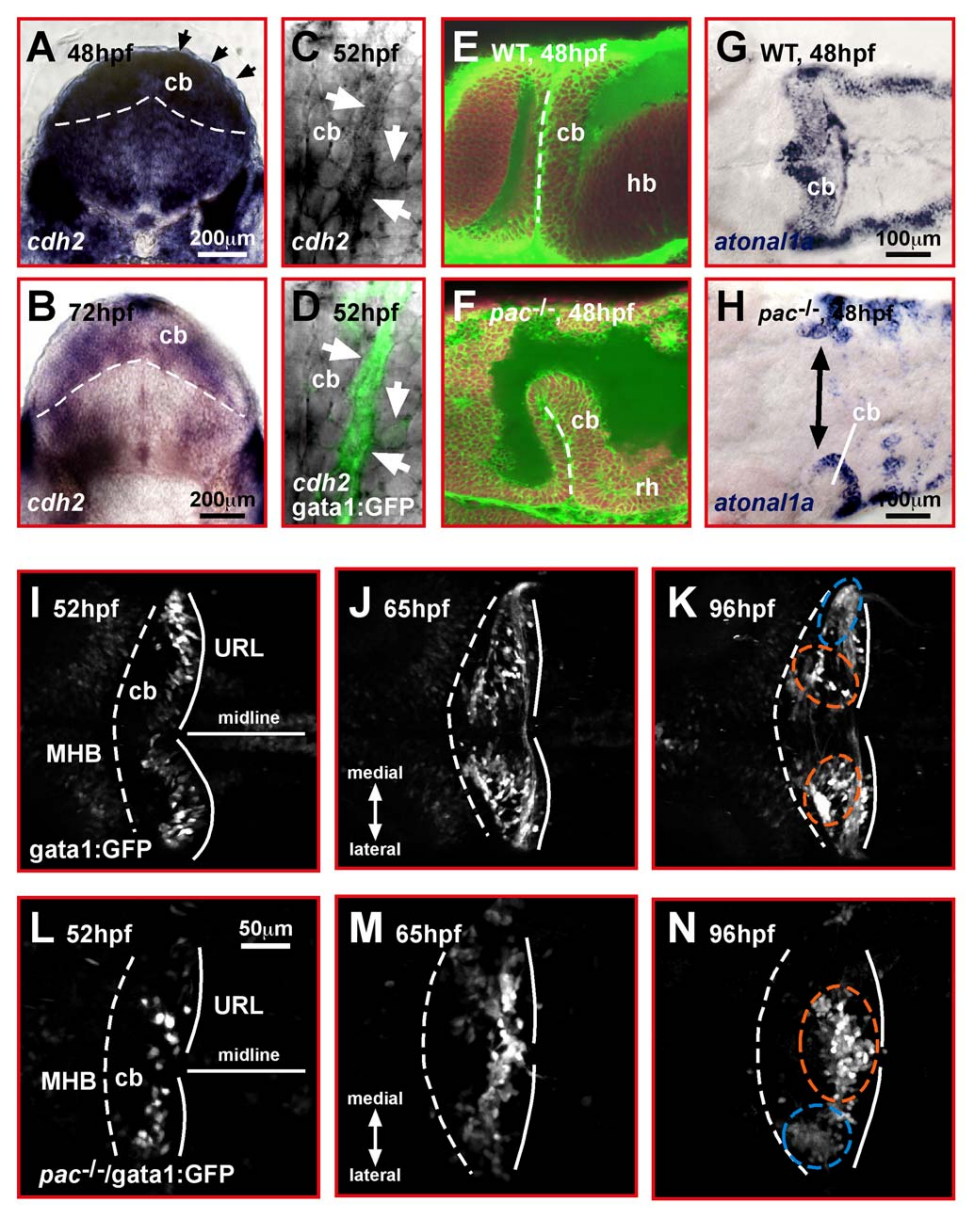
Cadherin-2 is a likely candidate to mediate GC migration in the zebrafish cerebellum. (A, B) Transverse view of the developing zebrafish cerebellum after ISH shows *cadherin-2* expression during stages of prominent GC migration (black arrowheads point out dorsal most cerebellar region where migrating GCs are positioned). (C, D) Lateral view high magnification of the cerebellum. Migrating GCs in gata1:GFP transgenic embryos co-express *cadherin-2* at high levels (white arrows). (E, F) Combined TOPRO and Bodipy-Ceramide staining of the cerebellum (lateral view) in WT (E) and *pac* ^−/−^ embryos (F). The rudimentary cerebellum in *pac* ^−/−^ embryos still forms a rhombic lip, as demarcated by expression of *atoh1a* (G: WT, H: *pac* ^−/−^ embryos, dorsal views, anterior is left). (I–N) Migration routes of GCs in the cerebellum. Dorsal view time-lapse sequence of migrating GCs in gata1:GFP transgenic embryos. (I–K) WT GCs migrate preferentially toward and along the MHB to form two clusters: the dorsomedial corpus cerebelli (orange dashed circles in K) and ventro-lateral eminentia granularis (blue dashed circle in K, see Video S2). (L–N) In *pac* ^−/−^ mutants, GCs of the dorsally forming corpus cerebelli remain close to the URL and settle in ectopic clusters (see Video S3). The MHB and URL are delineated with a dashed and solid line, respectively. Anterior is left. cb, cerebellum; hb, hindbrain; MHB, midbrain-hindbrain boundary; URL, upper rhombic lip.

The expression of *cadherin-2* has been well documented in zebrafish [7] as the only *cadherin* homolog expressed in regions of GC migration [23]. To confirm *cadherin-2* expression in the cerebellum during stages of prominent GC migration (between 48 and 96 hpf) we performed in situ hybridization (ISH) on transverse sections (Figure 2A, 2B). *cadherin-2* expression was particularly strong in dorsal-most cerebellar regions in domains where migrating GCs are localized (Figure 2A, black arrowheads). Furthermore, combined *cadherin-2* ISH and immunohistochemistry against GFP-expressing GCs in gata1:GFP embryos (Figure 2C, 2D, white arrowhead) revealed co-expression of *cadherin-2* in all GFP-positive GCs, often at higher levels than in GFP-negative neighboring cells. This shows that *cadherin-2* is expressed in zebrafish cerebellar GCs and it is a likely candidate to mediate homophilic interactions.

The zebrafish *parachute*R2.10 mutant (termed *pac* ^−/−^ from hereon) harbors an amorph loss of function allele of zebrafish *cadherin-2* [7]. Despite strong neurulation defects, homozygous *pac* ^−/−^ mutant embryos still develop a rudimentary cerebellar URL (Figure 2E, 2F), as defined by the expression of the rhombic lip marker gene *atonal1a* (Figure 2G, 2H).

To address whether Cadherin-2 functions in regulating cerebellar GC migration, we analyzed these mutants by in vivo time-lapse microscopy. GFP-expressing GCs in wild type (WT) gata1:GFP embryos start to migrate at 48 hpf from the URL anteriorly toward the MHB, where they turn laterally to settle in distinct clusters (Figure 2I–2K, dorsal view, see Video S2, *n* = 3). These clusters eventually differentiate into the GC populations of the eminentia granularis (Figure 2K, blue dashed circle) and the corpus cerebelli (Figure 2K, orange dashed circle) [25]. In contrast, time-lapse analysis of gata1:GFP/*pac* ^−/−^ mutant cerebella demonstrated that GCs, although being moderately motile, remained close to the URL during all stages analyzed (Figure 2L–2N). These cells moved over short distances along the medio-lateral aspect of the URL and failed to form their characteristic clusters at the MHB (see Video S3, *n* = 4). Consistent with previous findings in *pac* ^−/−^ mutants, lateral cells, such as GCs of the later forming eminentia granularis (blue dashed circle), were affected to a lesser extent than dorsal-most neuronal populations [7]. These findings suggest that Cadherin-2 is involved in regulating the highly ordered migration behavior of cerebellar GCs.

### Temporal Rescue of *Parachute* Mutant Embryos

The neurulation defects in homozygous *pac* ^−/−^-embryos result in smaller cerebellar lobes and a failure in dorsal midline closure (Figure 2F, 2H, black arrow). Because neurulation occurs early during CNS development, later defects in GC migration may be a secondary consequence. To test this possibility we temporally rescued the neurulation phenotype by injecting *cadherin-2*-mRNA (70 pg) into 1-cell stage embryos. Uninjected *pac* ^−/−^ embryos failed to fuse dorsal midline structures by 24 hpf (Figure S1B). In contrast, in most of the rescued embryos (72%, *n* = 39/54, termed *pac* ^−/−^R from here on) both cerebellar lobes aligned along the dorsal midline (Figure S1C) similar to WT cerebellar primordia (Figure S1A). The rescue of neurulation defects was further confirmed by the proper expression of *wnt1* along the dorsal midline in *pac* ^−/−^R embryos (Figure S1D–S1F). Similarly, dorsal *atonal1a* expression (Figure S1G) was largely restored in *pac* ^−/−^R embryos (Figure S1I, black arrowhead) when compared to *pac* ^−/−^-mutants (Figure S1H), although in some embryos deviations of lower rhombic lip tissue was found (Fig. S1I, white asterisk). Rescued *pac* ^−/−^R embryos were unambiguously identified as homozygous *pac* ^−/−^-carriers by single embryonic tail RT-PCR (Figure S1L) [7]. These findings indicate that the neurulation defects in *pac* ^−/−^-embryos can be rescued by *cadherin-2* mRNA-injections to largely restore normal cerebellar development.

Comparison of the presence of *cadherin-2* mRNA in *pac* ^−/−^ (Figure S1J) and *pac* ^−/−^R (Figure S1K) embryos by ISH revealed that the injected mRNA in *pac* ^−/−^R embryos was completely lost by 24 hpf, while Cadherin-2 protein levels were comparable to WT embryos (compare Western blot analysis in Figure S1M, lane 1 and 3, note: samples contain cytoplasmic and membrane fractions). Subsequently, Cadherin-2 levels declined in *pac* ^−/−^R embryos due to protein turnover and reached severely decreased levels at 48 hpf (Figure S1M, lane 6, black arrow), while undetectable by 72 hpf (Figure S1N). This demonstrates that Cadherin-2 protein is absent in *pac* ^−/−^R embryos during the major GC migration period between 48 and 96 hpf [25]. Thus the phenotypic rescue of *pac* ^−/−^R embryos is temporally restricted to developmental stages before the onset of GC migration and therefore allows for investigating the role of Cadherin-2 in directly regulating cerebellar GC migration. For all subsequent experiments, only *pac* ^−/−^R embryos with fused cerebellar midline structures at 48 hpf were used for analysis, thus verifying that neurulation defects had been unambiguously rescued.

### Cadherin-2 Mediates Directionality and Coherence of Cerebellar GC Migration

**Figure 3:**
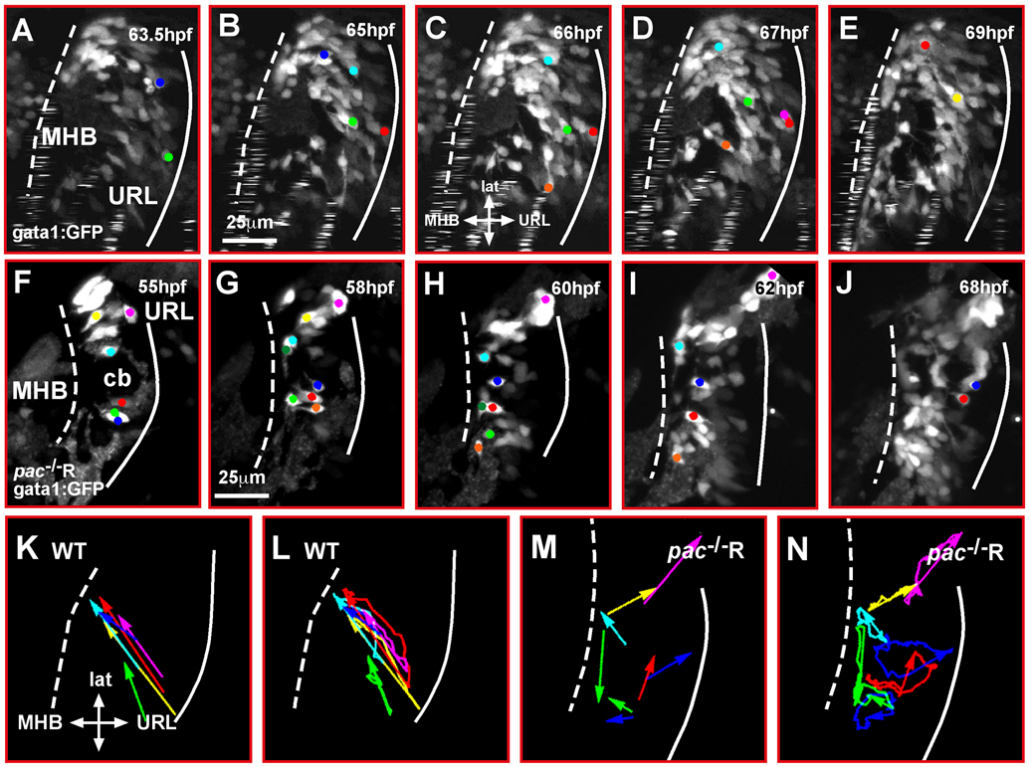
Cadherin-2 regulates coherence of cerebellar GC migration. (A–J) Maximum intensity projections of individual time-lapse stacks show GC migration in the right cerebellar lobe of gata1:GFP WT and gata1:GFP/*pac* ^−/−^R-embryos. Cell tracings reveal that GCs in WT cerebella (A–E) migrate collectively toward the MHB, whereas individually migrating *pac* ^−/−^R GCs (F–J) follow deviant paths (compare also Video S4 and Video S5). Plotting the migratory tracks from start to end point (K–N) shows a highly coherent and directional migration of WT GCs (K, L) in contrast to *pac* ^−/−^R GCs (M, N) (e.g., compare dark blue and red tracks in L and N). cb, cerebellum; MHB, midbrain-hindbrain boundary; URL, upper rhombic lip.

We next followed individual GCs in gata1:GFP/*pac* ^−/−^R embryos by intravital time-lapse confocal imaging. Cerebellar *pac* ^−/−^R GCs emigrated from the URL at about 55 hpf and migrated over longer distances (Figure 3F–3J), unlike *pac* ^−/−^-mutant GCs. In comparison to WT embryos however (Figure 3A–3E, Video S4, *n* = 5), these GCs migrated mostly as individual cells rather than interacting intensely within chains. More strikingly, individual *pac* ^−/−^R GCs deviated from their antero-lateral routes toward and along the MHB, migrating in all directions within the differentiating cerebellum (Video S5, *n* = 5).

These findings indicate that proper coordination of GC migration is aberrant in the absence of Cadherin-2, which became more apparent after comparing the migratory tracks of individual WT and *pac* ^−/−^R GCs. Tracks of WT GCs aligned almost parallel to one another (Figure 3K, 3L) as expected for the observed cohesive cell migration in chain-like structures. Also, the migratory routes of individual cells followed a strict antero-lateral path in linear direction from the URL to the MHB (Figure 3L), thus revealing that cerebellar GC migration in zebrafish is highly directional. In contrast, tracks of individual cerebellar GCs in *pac* ^−/−^R embryos were dispersed in a non-cohesive manner with many directional changes (Figure 3M, 3N, e. g., dark blue or red tracks). These findings demonstrate that Cadherin-2 regulates both cohesion and directionality of migratory cerebellar GCs in zebrafish.

### Cadherin-2 Is Required for Polarizing GCs Prior to Migration

**Figure 4:**
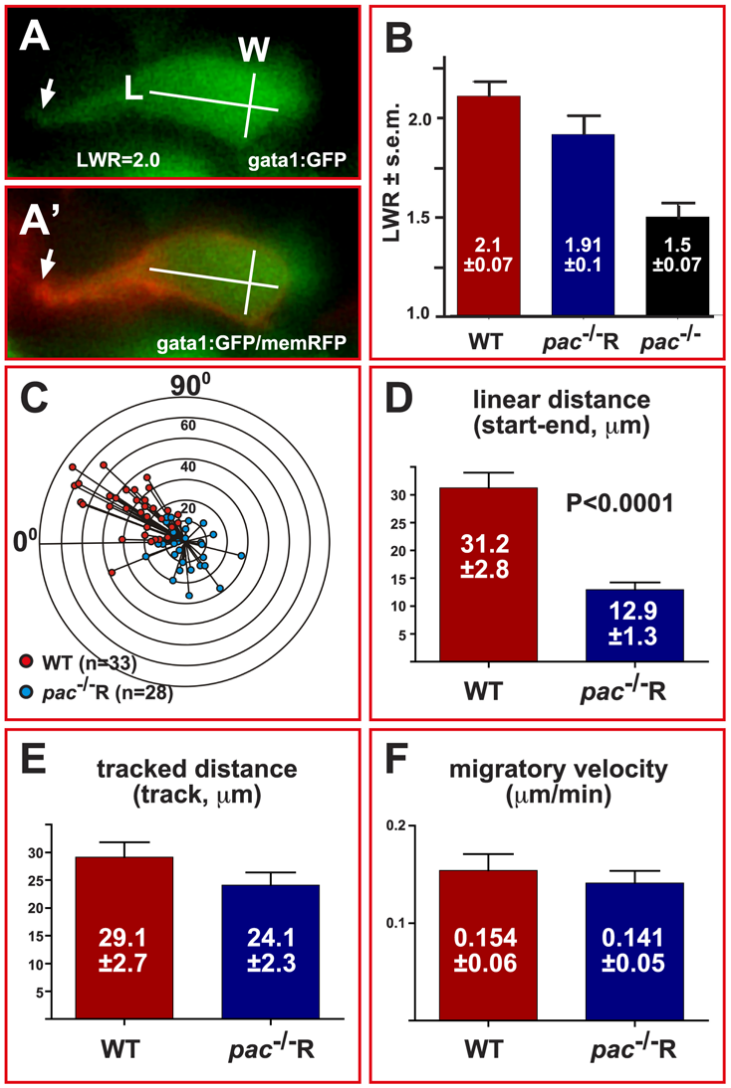
Cadherin-2 maintains directionality but not velocity of cerebellar GC migration. (A) Example of length-width measurements in individual gata1:GFP GCs, co-expressing membrane-bound RFP to visualize cell contours (A′, see also Video S6). (B) WT GCs (LWR 2.1±0.07, *n* = 50) and *pac* ^−/−^R GCs (LWR 1.91±0.1, *n* = 41) clearly polarize, unlike GCs in *pac* ^−/−^-mutants (LWR 1.5±0.07, *n* = 51). (C) Graph showing the migrated distance of GCs plotted against their direction over a time course of 4 hours (0° marks MHB, 90° marks lateral edge of cb, concentric circles indicate migration distance in 10 μm steps). The orientation of GCs in gata1:GFP-embryos (Figure 8C) correlates well with their migratory direction (red dots, *n* = 33). Similarly, the random orientation of GCs in gata1:GFP/*pac* ^−/−^R-embryos (Figure 8C) leads to random migration with no preferred direction (blue dots, *n* = 28). (D) The linear migration distance is dramatically reduced for *pac* ^−/−^R GCs (12.9±1.3 µm), compared to WT GCs (31.2±2.8 µm), while traced distances (E, WT: 29.1±2.7 µm versus *pac* ^−/−^R: 24.1±2.3 µm) and migratory velocities (F, WT: 0.154±0.06 µm/min versus *pac* ^−/−^R: 0.141±0.05 µm/min) are nearly identical. Error bars indicate standard errors of the mean (SEM).

To address the underlying cellular differences reflecting changes in migration behavior of *pac* ^−/−^ and *pac* ^−/−^R GCs we analyzed the cell shape of migratory GCs by determining their length-width ratios (LWRs). This ratio was obtained in dividing the longest dimension of the cell soma by the widest distance between the lateral cell walls (Figure 4A). These measurements were independent of GC orientation in the cerebellum. To first define whether GFP expression in the gata1:GFP line was sufficient to reliably outline cell contours, we co-expressed heat shock inducible membrane-targeted RFP (pBTol2-8xHSE:mem-RFP, injection: 15 ng/ml together with 2.5 ng/ml mRNA of Tol-transposase) in cerebellar GCs. Confocal time-lapse imaging and overlay of both fluorescent channels confirmed that GFP expression in gata1:GFP GCs reliably marks all cell compartments including the position of leading protrusions (Figure 4A, A′, white arrow, see also Video S6).

Migratory cells are usually considered polarized with LWR values above 1.7 [31]. Based on this value, migrating GCs in WT (LWR of 2.1±0.07, *n* = 50) and *pac* ^−/−^R-embryos (LWR of 1.91±0.1, *n* = 41) were clearly polarized (Figure 4B). In contrast, GCs in homozygous *pac* ^−/−^-embryos had a significantly lower LWR (1.5±0.07, *n* = 51), suggesting that they lack a prominent polarization similar to non-migrating terminally differentiated GCs (LWR of 1.28±0.16, *n* = 34, *p*<0.0001, unpublished data). These findings suggest an early function for Cadherin-2 in mediating cell polarization in the cerebellar neuroepithelium prior to GC migration. The probable loss of polarization in cerebellar GCs of *pac* ^−/−^-mutants likely results in the observed stationary behavior and aberrant positioning close to their place of origin (Figure 2L–2N).

### Cadherin-2 Acts Autonomously in Chains of Migrating GCs

**Figure 5:**
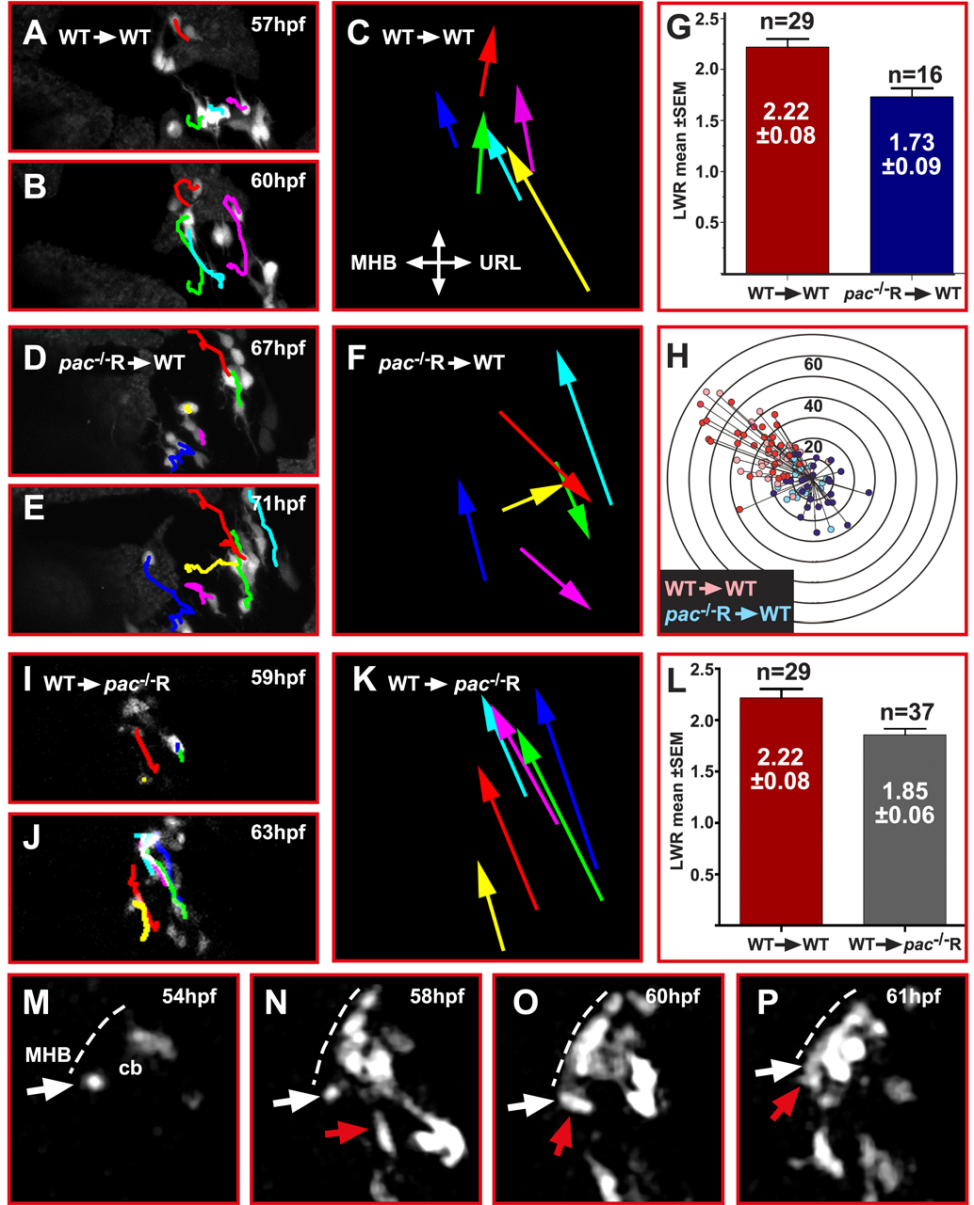
Cadherin-2 deficient GCs lack directional migration when transplanted into WT embryos. (A, B, D, E, I, J) Maximum intensity projections (dorsal view) of time-lapse recordings showing transplanted gata1:GFP/WT (A–C) and gata1:GFP/*pac* ^−/−^R (D–F) donor GCs in the right cerebellar lobe of a WT host, whereas (I, J) shows transplanted gata1:GFP/WT GCs in a *pac* ^−/−^R host cerebellum. Donor GCs derived from WT-WT transplantations are clearly polarized (G, red column, LWR: 2.22±0.08, *n* = 29) and migrate in a cohesive and directional manner, supported by cell tracing overlays (C) (see also Video S7, *n* = 4). In contrast, migration of *pac* ^−/−^R donor GCs in a WT cerebellum is non-cohesive and non-directional, despite polarization of these cells (G, blue column, LWR: 1.73±0.09, *n* = 16) (see also Video S8, *n* = 4). (H) Distance and migration direction of WT and mutant donor GCs (gata1:GFP/WT-WT: pink, *n* = 33; gata1:GFP/*pac* ^−/−^R: light blue, *n* = 18) in WT cerebella overlaid with tracings from respective non-transplanted GCs (see Figure 4C), i.e. gata1:GFP/WT (red) and gata1:GFP/*pac* ^−/−^R GCs (dark blue) to show a similar migration pattern in both respective groups (WT + WT-WT and *pac* ^−/−^R + *pac* ^−/−^R-WT. (I–K) Transplanted gata1:GFP/WT GCs migrate in a *pac* ^−/−^R host cerebellum in a cohesive and directional manner, supported by cell tracing overlays (see also Video S9, *n* = 3). (L) These Gata1:GFP/WT donors are polarized in a *pac* ^−/−^R mutant environment as indicated by their LWRs (WT-WT 2.22±0.08; *pac* ^−/−^R 1.85±0.06). (M–P) Transplantation of gata1:GFP/WT donor GCs into *pac* ^−/−^R hosts revealing directional chain migration of WT GCs in a *pac* ^−/−^R cerebellum. Note the group of GCs (red arrow in N, O, P) collectively migrating toward the MHB (dotted line) contacting an isolated quiescent GC, which subsequently joins the migrating GC chain (see also Video S9). All images are maximum intensity projections (dorsal view) of time-lapse recordings. Error bars indicate SEM. MHB: midbrain-hindbrain boundary.

We next asked whether the aberrant migration behavior of *pac* ^−/−^R GCs results from a cell-autonomous requirement for Cadherin-2 by generating chimeric embryos using a cell transplantation approach. Five to 20 cells were transplanted at the sphere stage (4 hpf, 4,000–8,000 cells) from either gata1:GFP WT or gata1:GFP/*pac* ^−/−^R donor embryos into WT hosts of the same developmental stage. Subsequently we analyzed the migration behavior of GFP-expressing GCs by in vivo time-lapse confocal microscopy. In the control experiment WT donor GCs transplanted into WT hosts followed linear migration routes toward the MHB (Figure 5A–5C, Video S7, *n* = 4) like GCs in WT embryos (Figure 5H). Transplanted gata1:GFP/*pac* ^−/−^R donor cells were polarized similar to non-transplanted *pac* ^−/−^R GCs (Figure 5G, LWR of 1.73±0.09, *n* = 16; Figure 4B), thus indicating their proper rescue by integration into the neuroepithelium.

In contrast to transplanted WT GCs however, *pac* ^−/−^R donor GCs migrated in the WT cerebellum along individual routes with many directional changes (Figure 5D–5F, Video S8, *n* = 4). Thus the migration behavior of transplanted Cadherin-2 deficient GCs was indistinguishable from cerebellar GCs in gata1:GFP/*pac* ^−/−^R embryos (Figure 5H, Figure 4C).

In the converse experiment WT gata1:GFP donor cells were transplanted into *pac* ^−/−^R hosts. The presence of Cadherin-2 in few donor cells was sufficient to allow chain formation and directional GC migration toward the MHB (Figure 5I–5K, Video S9, *n* = 3). Intriguingly we noticed isolated GFP-expressing GCs that paused in migration (Figure 5M, white arrow) for several hours, but upon contact with chain-migrating donor GCs (Figure 5O, red arrow) they joined the chain and continued migration (Figure 5P, Video S9). This shows that with respect to the migratory GC chains, Cadherin-2 acts in an autonomous manner and its expression is not required in other cerebellar neurons such as eurydendroid or Purkinje neurons [32] for proper GC migration.

### Cadherin-2 Mediates Cell-Cell Interactions in Migratory Chains of Zebrafish GCs

**Figure 6:**
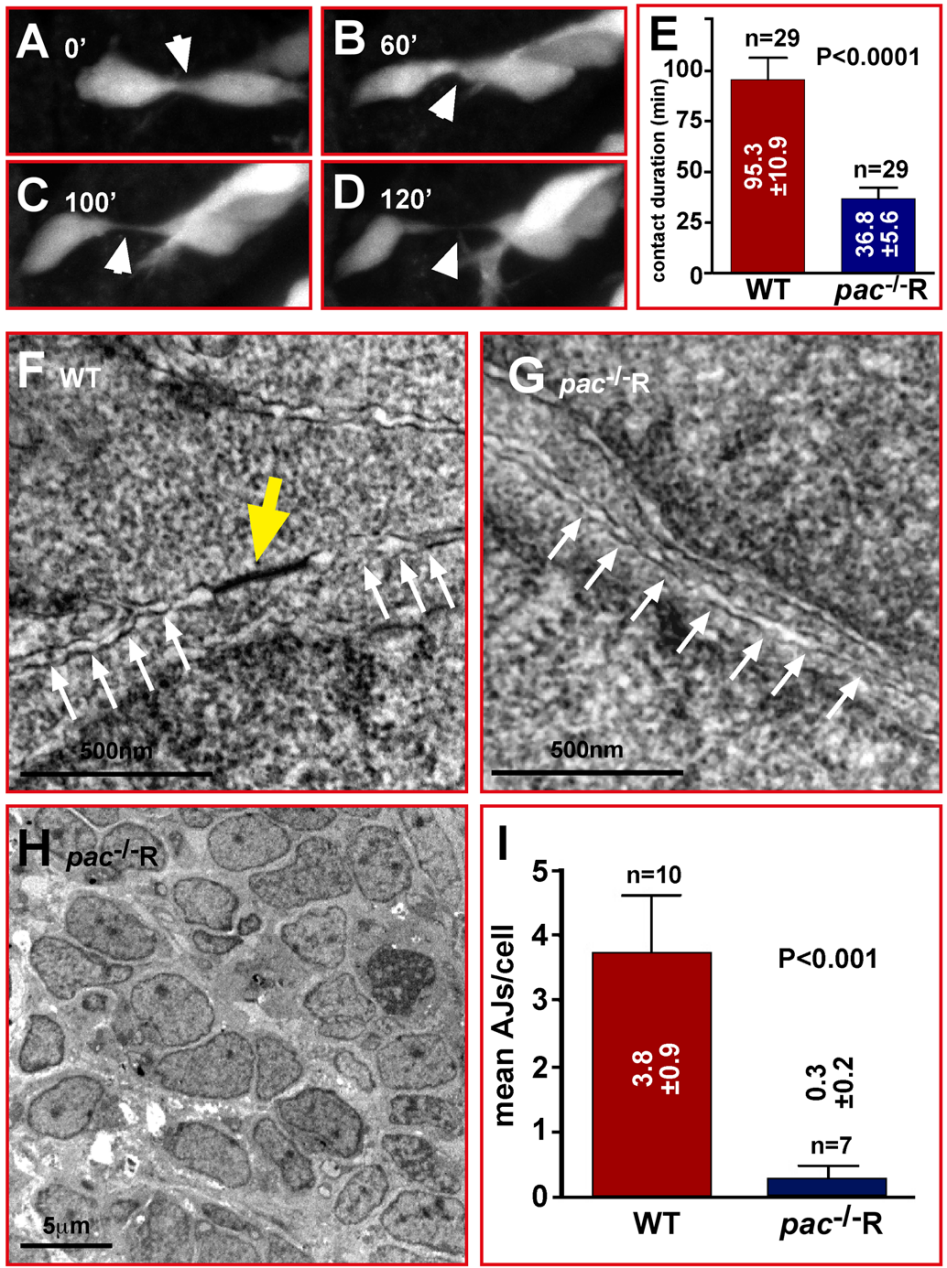
Migratory *pac* ^−***/*−**^ **R cerebellar GCs show reduced contact stability and adherens junctions.** (A–D) Comparison of contact duration between individual gata1:GFP GCs in time-lapse sequences (white arrowheads) reveals that lack of Cadherin-2 in *pac* ^−/−^R GCs significantly reduces the duration of stable cell-cell contacts (E, WT: 95.3±10.9 min versus *pac* ^−/−^R: 36.8±5.6 min, *n* = 29). (F–H) TEM analysis reveals that AJs between *pac* ^−/−^R cerebellar neurons are largely absent (G), while numerous AJs are found between individual WT cerebellar neurons (F). (H) *pac* ^−/−^R cerebellar neurons appear disorganized (compare with Figure 1J). (I) Quantification of TEM images (3.8±0.9 AJs, *n* = 10, in WT versus 0.3±0.2 AJs, *n* = 7, in *pac* ^−/−^R-cerebella).

Two effects on migration were observed in GCs lacking Cadherin-2: impaired cohesion and a failure in maintaining directionality. In order to better understand the lack of cohesion between migrating cerebellar *pac* ^−/−^R GCs, we quantified the stability of cell-cell contacts between GC pairs. In WT gata1:GFP transgenic embryos, contacts between two GCs (Figure 6A–6D, white arrowhead) lasted for an average duration of 95.3 min (Figure 6E, *n* = 29 analyzed cell contacts). In contrast, GC contacts occurred less often in *pac* ^−/−^R embryos and lasted significantly shorter, on average 36.8 min (Figure 6E, *n* = 29 analyzed cell contacts, *p*<0.0001). This is shorter than the duration of one forward moving step of migrating GCs, which takes about 45 to 60 min.

We next performed ultrastructure analysis of *pac* ^−/−^R cerebella by transmission electron microscopy to analyze the presence of adherens junctions. We noticed a highly disorganized cellular arrangement in the cerebellum of *pac* ^−/−^R embryos (Figure 6H, compare to Figure 1J). Furthermore, compared to WT embryos in which prominent adherens junctions were visible (Figure 6F, yellow arrow) barely any could be detected in the cerebellum of *pac* ^−/−^R embryos (Figure 6G, white arrows mark cytoplasmic membrane between adjacent cells). The significant reduction or even absence of adherens junctions in the cerebellum of *pac* ^−/−^R embryos was confirmed when their numbers per cell were quantified in comparison to WT counterparts (WT: 3.8±0.9, *n* = 10, versus *pac* ^−/−^R: 0.3±0.2, *n* = 7, *p*<0.001, Figure 6I).

### GCs Require Cadherin-2 to Join Chains of Migrating GCs

**Figure 7:**
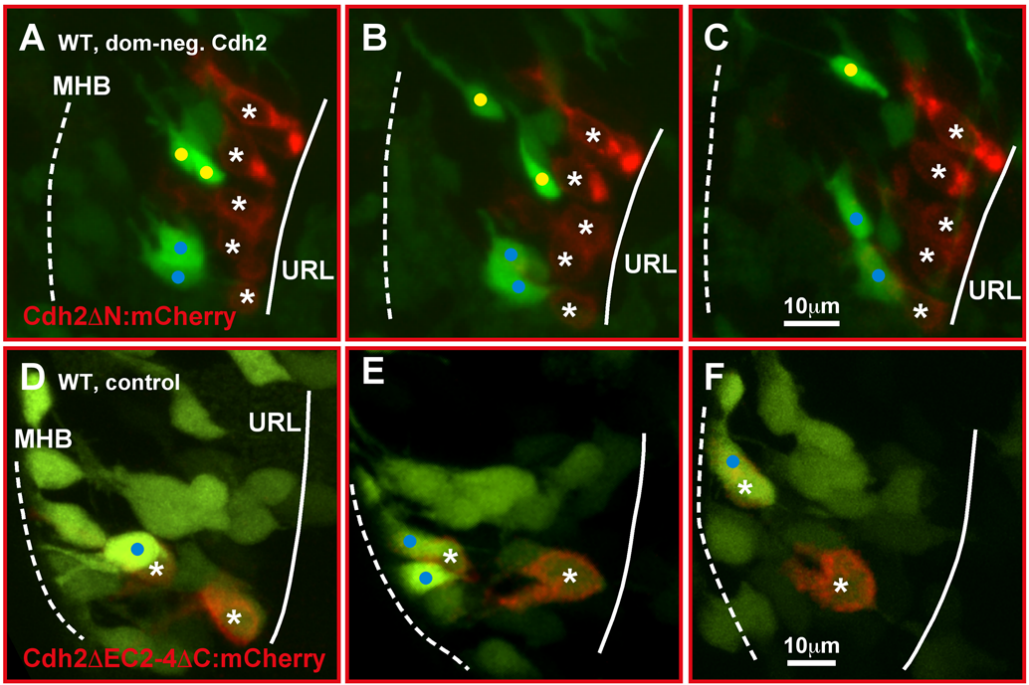
Inhibition of Cadherin-2 function impairs emigration of URL progenitor cells. (A–C) Maximum intensity projections of time-lapse movie (dorsal view) showing GFP-negative granule progenitors in the URL (solid line) of gata1:GFP embryos expressing a dominant-negative Cadherin-2 variant under the control of a heatshock-inducible promoter element at 55 hpf (8xHSECdh2ΔN-mCherry). Fluorescently labeled progenitors fail to delaminate from the URL and do not join GC chains migrating toward the MHB (dotted) (see Video S10). (D–F) Expression of Cdh2ΔEC2–4ΔC-mCherry, a non-functional Cdh2-reporter, does not interfere with GC migration. These GCs co-activate GFP during initiation of migration toward the MHB (see Video S11) [25]. MHB, midbrain-hindbrain boundary; URL, upper rhombic lip.

The reduced contact duration and number of adherens junctions in *pac* ^−/−^R GCs suggested that *pac* ^−/−^R URL cells are not able to adhere to forming migratory GC chain-like structures. We therefore tested whether the disruption of Cadherin-2 mediated adhesion in individual URL cells impairs their ability to join migratory GC chains emanating from the URL. To temporally affect the function of endogenous Cadherin-2, we induced a dominant-negative variant of zebrafish Cadherin-2 Cdh2ΔN fused to mCherry (Figure S2A) [33], under control of an oligomerized heat shock consensus sequence [34] from vector pB8xHSE:Cdh2ΔN–mCherry at 55 hpf. Strongly red fluorescent URL cells (Figure 7A, white asterisks) retained their polarization and oriented in an antero-lateral direction toward the MHB. Nevertheless time-lapse analysis revealed that these URL cells failed to join chains formed by neighboring GFP-expressing emigrating WT GCs (Figure 7A–7C, migrating GC chains marked with yellow or blue dots, Video S10, *n* = 4).

As control, strong expression of a membrane-targeted but non-functional Cadherin-2 deletion variant Cdh2ΔC2-4ΔC fused to the fluorescent protein mCherry (Figure S2A and below) at 55 hpf was induced by heat shock. Expression of this variant (Figure S2A and below) resulted in strongly red fluorescent URL cells but did not affect their onset and proper migration together with WT gata1:GFP GCs (Figure 7D–7F, white asterisks, Video S11, *n* = 5). Taken together these findings show that Cadherin-2 is the predominant classic Cadherin in migrating zebrafish GCs that is required to convey homophilic cell-cell interactions for cohesive GC migration toward the MHB.

### Impaired Homophilic Cadherin-2 Interactions Do Not Affect GC Motility

Because of the reduced contact duration among *pac* ^−/−^R GCs we wondered whether lack of Cadherin-2 expression would also affect the motility of migrating GCs. We therefore traced individual GCs en route to the MHB. First, we measured the linear distance that GCs migrated over a four-h period and plotted this distance (µm) as a function of the direction of migration in the cerebellum. This analysis showed that GCs in WT embryos consistently migrated toward antero-lateral regions at the MHB (Figure 4C, *n* = 30/33, 91%, red dots). In contrast, cerebellar GCs in *pac* ^−/−^R embryos did not display a preferred directionality. In fact, more than one third of the analyzed cells (Figure 4C, *n* = 11/28, 39%, blue dots) migrated posteriorly toward the URL. Intriguingly, quantification of the linear distances of migrating GCs showed that WT cells migrated almost three times as far as their *pac* ^−/−^R counterparts (Figure 4D, 31.2 μm versus 12.9 μm, *p*<0.0001). However, the total length of the migratory routes (Figure 4E, 29.1 µm versus 24.1 µm) and the average migratory velocities (Figure 4F, 154 nm/min versus 141 nm/min) were nearly identical. The signature of the migratory routes of GCs in pac^−/−^R embryos can explain these discrepancies in linear and total length of migration. GCs in WT embryos migrate almost linearly in the direction of their polarization (Figure 3L, Figure 4C), while *pac* ^−/−^R GCs frequently change directions (Figure 3N, Figure 4C), sometimes even displaying circling behaviors (e.g., dark blue marked cells). These findings show that the lack of Cadherin-2 does not cause a reduction in GC motility but suggests a polarity defect for *pac* ^−/−^R GCs being unable to maintain a specific orientation while migrating at normal velocities along curved routes.

### Cadherin-2 Maintains the Directionality of Cerebellar GCs during Migration

**Figure 8:**
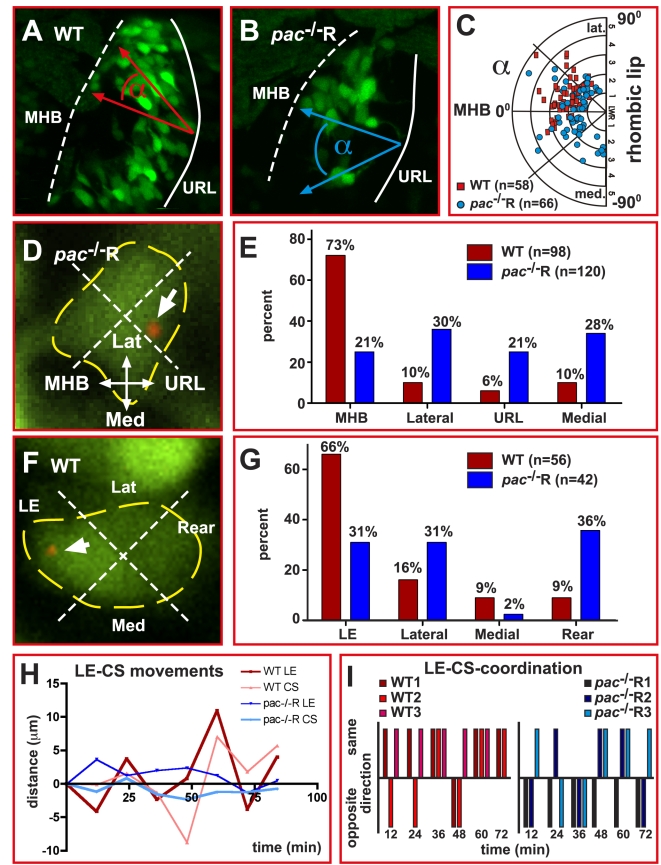
Centrosome positioning is uncoupled from migratory directionality in Cadherin-2 deficient GCs. (A, B) Example for quantification of GC polarization using dorsal view confocal projections of right cerebellar lobes in WT (A) and *pac* ^−/−^R (B) gata1:GFP embryos. For quantification, the polarization of GFP-expressing GCs (LWR see Figure 4B) as well as the orientation of the long axis of a GC with respect to the MHB was determined. (C) The angle between the long axis of a GC and the linear axis between MHB and URL is plotted as function of their respective LWRs. Whereas WT GCs preferentially polarize in anterior-lateral directions toward the MHB (red squares, *n* = 58), *pac* ^−/−^R GCs display no preference in polarization, being randomly oriented in the cerebellum (blue dots, *n* = 66). (D, F) Method for quantification of centrosome position in GCs co-expressing the centrosome-localized centrin-td-Tomato fluorescent protein (white arrows mark centrosome, yellow dashed lines mark soma). (E) While the centrosome in more than 70% of analyzed WT GCs (red bars, *n* = 98) is localized toward the MHB, the centrosome in *pac* ^−/−^R GCs shows no preferred orientation (blue bars, *n* = 120). (F) Quantification of centrosome position with respect to cell morphology. (G) In WT GCs, the centrosome preferentially locates to the leading edge, while no preferred location is found in *pac* ^−/−^R GCs. (H, I) The relationship between centrosome and leading edge dynamics was quantified by simultaneously tracing these structures within the same GC (*n* = 3, each group), using the ImageJ manual tracking tool. (H) In WT GCs, the centrosome movements follow leading edge movements thus moving in the same direction (I, *n* = 13/17, see Video S12), whereas in *pac* ^−*/*−^R GCs centrosome movements independent of the leading edge are apparent often occurring in opposite directions (I, *n* = 7/18 movements in same direction, see Video S13). Lat, lateral; LE, leading edge; Med, medial; MHB, midbrain-hindbrain boundary; URL, upper rhombic lip.

In order to investigate the underlying mechanisms leading to Cadherin-2 mediated directional migration of cerebellar GCs, we first analyzed whether GCs were polarized in direction of migration. For this, we determined the angle between the antero-posterior axis of the cerebellum and the long axis of GCs and plotted this angle, which depicts a neuron's orientation, as a function of its respective LWR (Figure 8A–8C). We found that although WT and *pac* ^−/−^R GCs were clearly polarized (Figure 4B), their orientation of elongation differed. WT GCs were preferentially oriented toward antero-lateral directions (0–90°, Figure 8C, *n* = 58, red dots), consistent with future locations of cerebellar GC clusters in medial and lateral positions close to the MHB (Figure 2K). In contrast, the orientation of *pac* ^−/−^R GCs was random and did not reveal any preference in their orientation, with angles covering almost the entire 180 degrees (Figure 8C, *n* = 66, blue dots).

Cerebellar GCs migrate via nucleokinesis with the centrosome positioned in front of the nucleus and facing toward the leading edge of the cell. We used this property of the centrosome as an intrinsic polarity marker to determine whether migrating gata1:GFP GCs in *pac* ^−/−^R cerebella orient their centrosome into direction of migration. To image the position of the centrosome, we expressed Centrin2:tdTomato (pCS-GalTA and pBU-Centrin2:tdTomato) in gata1:GFP GCs (Figure 8D, 8F). First, we analyzed the centrosome position with respect to cerebellar morphology by subdividing GCs into quadrants facing toward the MHB, the URL, as well as the medial and lateral region of the cerebellum (Figure 8D). In 73% (*n* = 72/98) of analyzed WT GCs the centrosome positioned anteriorly toward the MHB, consistent with the migratory direction of these cells (Figure 4C). Strikingly, in GCs of *pac* ^−/−^R embryos the centrosome did not display any favored orientation and pointed almost equally toward the MHB (21%, *n* = 25/120), the lateral edge (30%, *n* = 36/120), the URL (21%, *n* = 25/120), or the midline (28%, *n* = 34/120) of the cerebellum (Figure 8E).

Next, we analyzed the position of the centrosome with respect to GC morphology but irrespective of the cell's orientation in the cerebellum by subdividing GCs into quadrants facing toward the leading edge, the rear, or the lateral sides of the cell (Figure 8F). In the majority of analyzed WT GCs the centrosome was positioned toward the leading edge (66%, *n* = 37/56) and was seldom found close to the rear of the cell (9%, *n* = 5/56, Figure 8G). In contrast, the centrosome in *pac* ^−/−^R GCs was positioned close to the leading edge in only 31% of analyzed GCs (*n* = 13/42) and faced in a similar number of cells to the rear (36%, *n* = 15/42, Figure 8G). These results indicate a coupling of leading edge and centrosome movements in GCs during migration, which appears to be lost in Cadherin-2 deficient GCs. This was further supported when leading edge and centrosome movements were followed by time-lapse recording and traced in vivo (*n* = 3). While in WT GCs the centrosome mostly followed the movements of the leading edge (Figure 8H, 8I, WT *n* = 13/17 movements in same direction, see also Video S12), centrosome movements occurred independent of the leading edge in *pac* ^−/−^R GCs often in opposite direction (Figure 8H, 8I *pac* ^−/−^R *n* = 7/18 movements in same direction, see also Video S13). Taken together these results indicate a defect of *pac* ^−/−^R GCs in maintaining polarity, which could be explained by an uncoupling of leading edge and centrosome movement in the absence of Cadherin-2, thus resulting in the failure of GCs to maintain directionality.

### Cadherin-2 Is Relocalized in Migrating Cerebellar GCs

The function of Cadherin-2 in regulating coherence and directionality of GC migration suggested a specific subcellular localization for Cadherin-2. To visualize Cadherin-2 directly in migrating GCs, we initially expressed a full-length Cadherin-2-GFP fusion by electroporation of expression constructs into the URL of WT embryos. The resulting overexpression of Cadherin-2 caused an arrest of GC migration. We therefore decided to generate an improved Cadherin-2 reporter by replacing the intracellular C-terminus of Cadherin-2 with the fluorescent protein mCherry, thereby disrupting Cadherin-2 interactions with the cytoskeleton. In addition, we removed the ectodomains 2–4 to allow weak cis- but not trans-dimerization of Cadherin-2 [13]. This variant, termed Cdh2ΔEC2-4ΔC-mCherry, when ectopically expressed, affected neither embryonic development (Figure S2A, S2D–F) nor GC migration (Figure 7D–7F). It however reliably co-localized with a full-length Cdh2-GFP fusion protein (Figure S2G–S2I) and furthermore directly interacted with Cadherin-2 as shown in co-immunoprecipitation experiments (Figure S2J). In addition, this variant localized in clusters at cytoplasmic membranes of dorsal cerelleber cells as evidenced by counterstaining with the membrane-dye Bodipy Ceramide (Figure S2K–S2M, green dashed oval). We therefore used this variant as an in vivo indicator of Cadherin-2 localization in migrating GCs.

**Figure 9:**
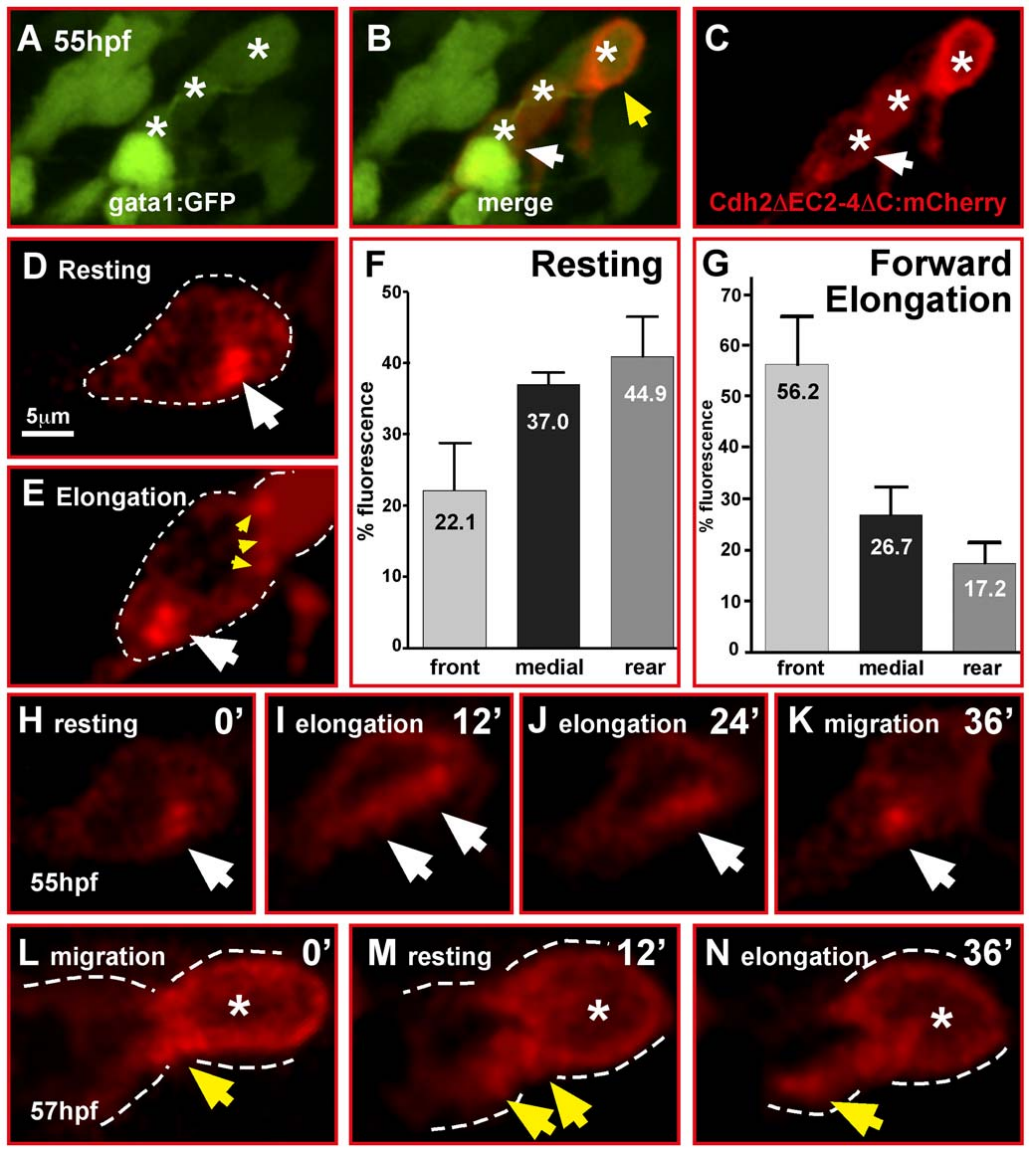
Cadherin-2 relocalization is coordinated with GC migration. (A–E, H–N) Single optical sections of time-lapse recordings using laser-scanning confocal microscopy. (A–C) Heat shock-induced expression of Cadherin-2 reporter protein (Cdh2ΔC2-4ΔC:mCherry) in a cerebellar GC chain (white asterisks). White arrows in (B, C) mark the neuron shown in (H–K), see also Video S14. (D, E) Cdh2ΔC2-4ΔC:mCherry fluorescence is localized in clusters in contact with neighboring GCs (E, yellow arrowheads), but cluster localization changes during migration mostly differing during resting (D) and forward elongation (E) of GCs prior to migration. (F) Quantification of mCherry-fluorescence shows that in resting GCs Cadherin-2 clusters are more evenly distributed, but preferentially localized in the medial and rear compartment of the cell (*n* = 5, *p* = 0.056, error bars indicate SEM). (G) Forward migration, in contrast, leads to Cadherin-2 redistribution towards the front compartment of GCs (*n* = 5, *p*<0.01). (H–K) Individual images of a time-lapse movie demonstrating the relocation of the Cdh2ΔC2-4ΔC:mCherry reporter (white arrow) after a resting phase from the rear of the cell (H) along the cytoplasmic membrane (I, J) during elongation of the GC to the front (K) to prepare for the next forward movement. (L–N) Localization of Cdh2ΔEC2-4ΔC-mCherry reporter in GCs contacting each other in a migratory GC chain (B, yellow arrowhead). (L) Fluorescence is condensed at contact sites of GCs but partly being redistributed according to migratory movements (M, N).

We transiently expressed this Cadherin-2 reporter (pB8xHSE:Cdh2ΔEC2-4ΔC-mCherry, heat shock induction at 50 hpf) in gata1:GFP GCs and imaged its behavior in double-fluorescent GCs during chain migration (Figure 9A–9C, white asterisks) by high magnification in vivo time lapse microscopy (Figure 9B, 9C, red fluorescent GC marked with white arrowhead is being followed in Video S14, *n* = 4). In this analysis we found that Cadherin-2 clusters were mostly localized in close apposition to neighboring cells as expected for adherens junctions (Figure 9E, yellow arrowheads; see also an overlay of green and red channels in Video S14).

Cerebellar GCs migrate via nucleokinesis, which involves alternating phases of resting periods followed by forward elongation and movement. To test whether Cadherin-2 is localized to a specific cell compartment during any of these phases, we quantified the percentage of mCherry fluorescence in the front, medial, and rear third of individual GCs, expressing the Cadherin-2 adhesion reporter construct. In resting GCs, mCherry-fluorescence was more equally distributed throughout the cell with a tendency to accumulate in the rear compartment (Figure 9D, 9H, white arrow). On average the rear compartment contained about 45% of total fluorescence, while only 22% of the fluorescence was localized in the front compartment (Figure 9F, *n* = 5, *p* = 0.056). In contrast, elongating GCs in preparation of forward migration contained on average 56% of mCherry-fluorescence in the front compartment (Figure 9E, 9K, white arrow) as opposed to only 17% in the rear (Figure 9G, *n* = 5, *p*<0.01, see also Video S14, yellow arrowhead marks decreasing fluorescence in rear). These observations suggest that Cadherin-2 mediated adhesion is redistributed during GC migration in coordination with GC movements (Figure 9H–9K).

To coordinate this movement within a migratory chain, Cadherin-2 must remain in contact with co-migrating GCs. We therefore followed the localization of the Cdh2ΔEC2-4ΔC-mCherry reporter also in GCs in trailing positions (Figure 9B, yellow arrowhead). During GC forward movement the mCherry reporter was localized mainly to the front of the GC at contact sites to the leading but resting GC (Figure 9L). When both cells rested, the reporter began to disassemble from this site of cell-cell contact but never completely disappeared (Figure 9M, yellow arrowhead). Subsequently, during elongation of the leading GC preparing for migration, fluorescence of the mCherry reporter was shifted within this cell towards the front compartment (Figure 9N, yellow arrowhead), with some of the reporter remaining in contact with the trailing cell.

**Figure 10:**
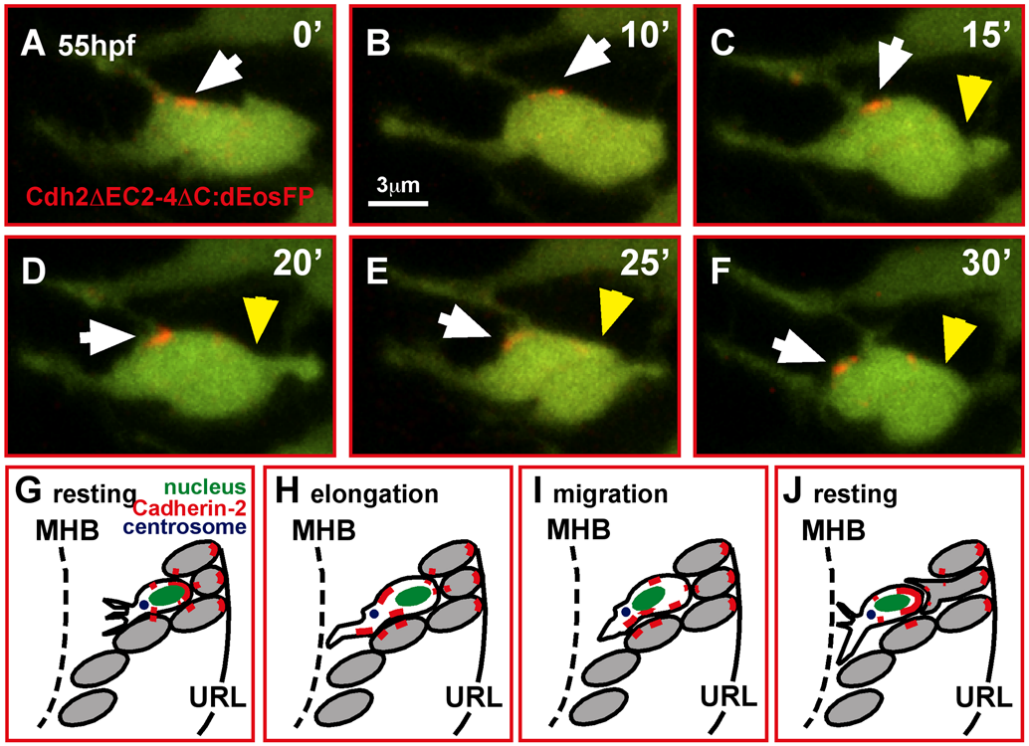
In migrating GCs Cadherin-2 is relocalized along the membrane. (A–F) Time-lapse sequence of Cdh2ΔEC2-4ΔC:dEosFP clusters, UV-converted at the membrane of a migrating GC in a gata1:GFP embryo (white arrowheads). A photo-converted cluster (red) moves successively toward the leading edge during GC elongation and migration (yellow arrowheads mark the trailing edge of the cell, see also Video S15). (G–J) Model of Cdh2-regulated granule cell migration (see text). MHB, midbrain-hindbrain boundary; URL, upper rhombic lip.

The initially observed relocation of clusters of the Cadherin-2 reporter consistent with migratory movements of GCs suggested that this process might in part be regulated by Cadherin-2 transport along the cytoplasmic membrane. This fluorescence quantification data have to be interpreted cautiously though as the reporter may not entirely indicate the proper endogenous expression of Cadherin-2. In addition, localization of the reporter in organelles of the secretory pathways like the Golgi may influence the quantification results, as these organelles are dynamic as well. In order to better understand how the position of Cadherin-2 clusters in the cytoplasmic membrane of migrating GCs is dynamically regulated during GC migration, we replaced mCherry with a UV-convertible fluorescent protein dEosFP, a homodimerizing pseudo-monomer [35]. We transiently expressed this construct (8xHSE: Cdh2ΔEC2-4ΔC-dEosFP) in gata1:GFP GCs and converted this reporter from green to red fluorescence in a small region of the cytoplasmic membrane using focal 405 nm excitation. When time-lapse imaging small clusters of the photoconverted Cadherin-2 variant (Figure 10A–10F, white arrowhead, see Video S15, *n* = 3) we found that during GC forward migration Cdh2ΔEC2-4ΔC:dEosFP remained strictly within the cytoplasmic membrane during translocation toward the front compartment within a 30 min interval (Figure 10C–10F, note the elongating trailing edge marked by yellow arrowhead, n = 4).

Taken together Cadherin-2 relocation appears to be coordinated with GC migration along the cytoplasmic membrane to ensure proper migration of GCs. Based on these observations we propose that during resting phases of GC migration Cadherin-2 mediated adhesion is strong at the rear of the cell and weaker in the front compartment (Figures 9H, 10G), probably allowing for flexible pathfinding of the leading edge. During GC forward elongation, Cadherin-2-mediated adhesion relocates to the front of the GC (Figures 9I–9K, 10H), while the centrosome, the nucleus (Figure 10H, 10I), and eventually the cytoplasm follow, likely facilitated by a decrease of adhesion in the cell's rear. When GCs reach their new position, the majority of Cadherin-2 is relocated again into the rear compartment (Figure 10J) until the next migratory step occurs.

### Impaired Directional Migration Affects the Terminal Differentiation of GCs

**Figure 11:**
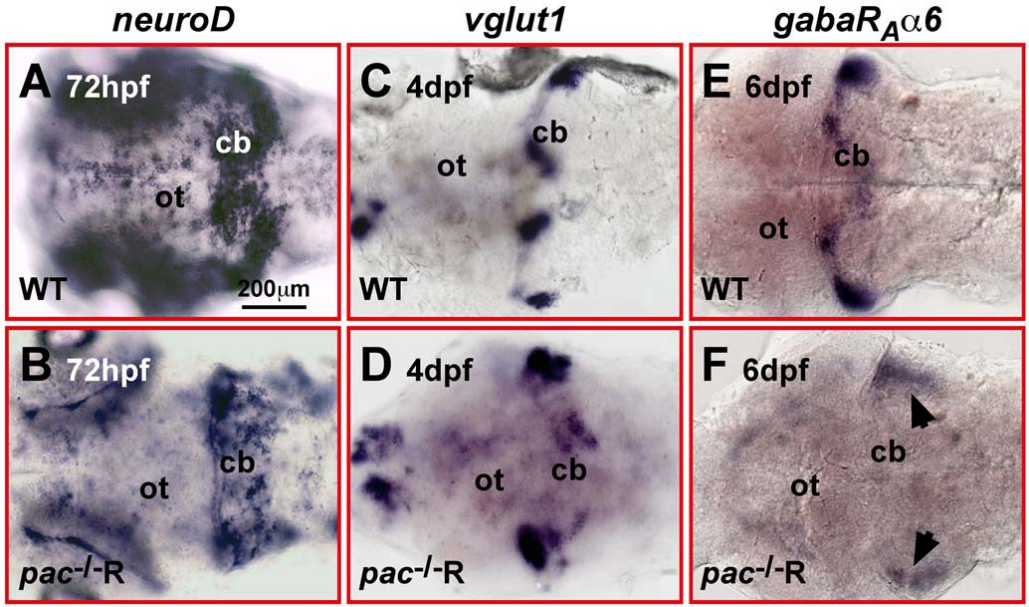
Impaired terminal differentiation of migrating Cadherin-2 deficient GCs. (A–F) Dorsal views of WT (A, C, E) and *pac* ^−/−^R cerebella (B, D, F). ISH for the granule cell differentiation markers *neuroD* (A, B, 3dpf), *vglut1* (C, D, 4dpf), and *gabαRa6* (E, F, 6dpf). *NeuroD* expression in *pac* ^−/−^R cerebella appears scattered, likely resulting from randomly migrating GCs. However, the strength of expression is similar to WT. *Vglut1* expression is strongly reduced in *pac* ^−/−^R and expression of the terminal granule cell differentiation marker *gabaR_A_α6* is almost absent in *pac* ^−/−^R cerebella (black arrows depict residual patches of expression in ventro-lateral cerebellar regions). cb, cerebellum; ot, optic tectum.

Because of the aberrant migration and final positions of Cadherin-2 deficient GCs, we wondered whether their differentiation is affected in *pac* ^−/−^R embryos. We first analyzed the expression of several GC differentiation markers at different developmental stages. At 72 hpf, the cerebellar GC population strongly expressed *neuroD*, a marker for differentiating postmitotic cerebellar GCs [25],[36], in both WT (Fig. 11A) and *pac* ^−/−^R embryos (Figure 11B). However in the latter, *neuroD*-expression was non-homogeneous and scattered throughout the dorsal cerebellum, consistent with their impaired directional migration behavior. At 4 dpf, the expression of the *vesicular glutamate transporter 1*, *vglut1*, could be detected in *pac* ^−/−^R cerebella, however the medial expression domains were weak and aberrantly located (Figure 11D) as compared to *vglut1* expression in WT (Figure 11C). This impairment in terminal GC differentiation was further supported by the complete absence of the expression of *vglut1* (unpublished data) and the specific cerebellar granule neuron marker *gaba_A_ receptor alpha6*-subunit, *gaba_A_Rα6*, in dorsal regions of the cerebellum in *pac* ^−/−^R embryos at 6 dpf (compare Figure 11E, 11F) [25]. The small remaining patches of *gaba_A_Rα6*-expression in more ventro-lateral regions (Figure 11F, black arrows) may reflect previous observations that ventral populations are affected to a lesser extent by the loss of Cadherin-2 in homozygous *parachute* embryos [7], similarly to our observations for GCs of the eminentia granularis (Figure 2, L–N blue dashed circle and Video S2). These findings indicate that improperly migrating Cadherin-2-deficient GCs initiate to differentiate, but they fail to terminally differentiate into mature cerebellar granule neurons.

**Figure 12:**
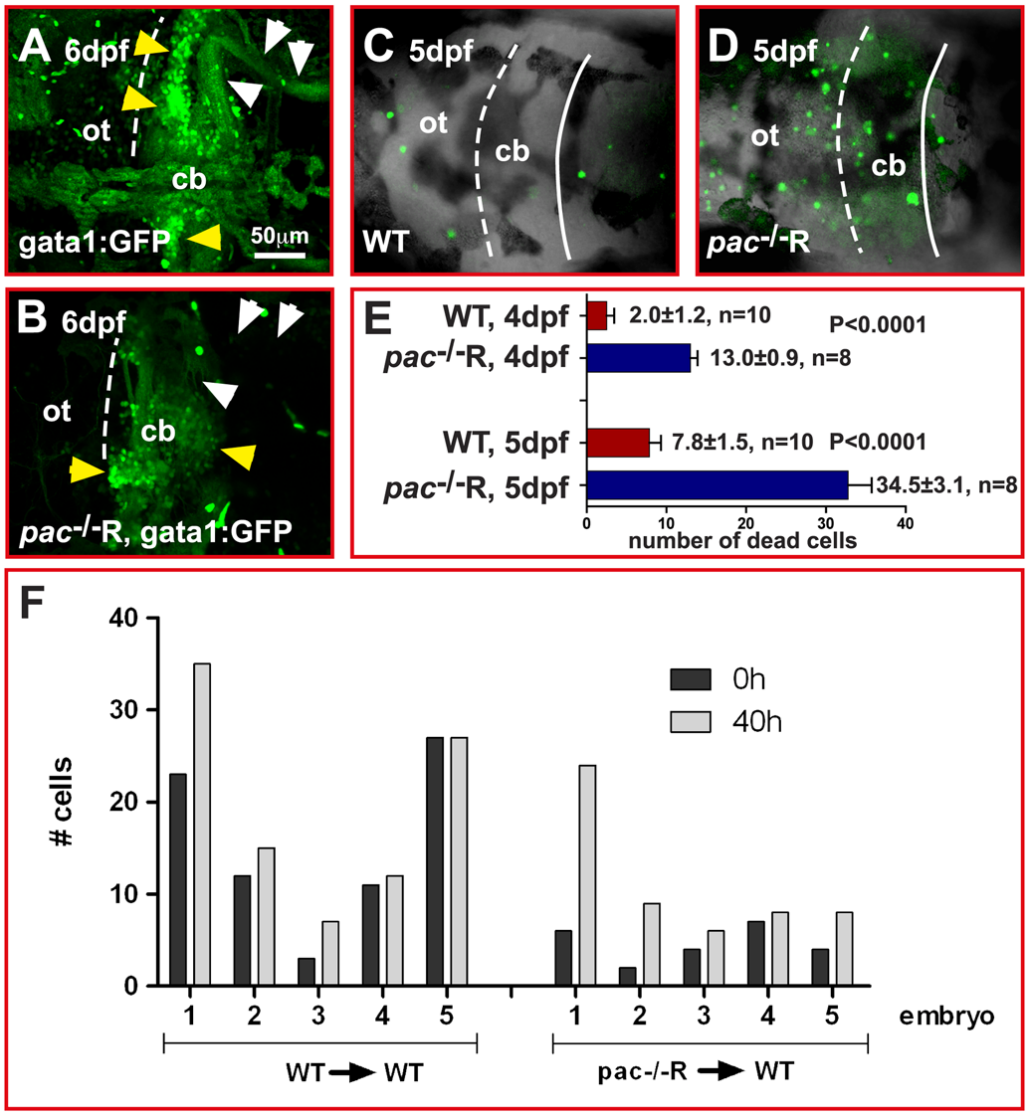
Increased cell death in *pac* ^−/−^R cerebella at 5 dpf. (A–D) Dorsal views of confocal image stacks showing projected gata1:GFP WT (A) and *pac* ^−/−^R (B) cerebella. Comparison indicates that the number of GFP-expressing GCs (yellow arrowheads) is severely reduced in *pac* ^−/−^R and parallel fiber projections (white arrowheads) are lacking, consistent with a strong increase in cell death as visualized by acridine orange staining (compare WT: C to *pac* ^−/−^R: D). (E) Quantification reveals that cell death in *pac* ^−/−^R increases 3-fold between 4 and 5 dpf (after GC migration has ceased). For analysis, the student *t*-test was used, ***p<0.0001. Error bars indicate SEM. (F) Quantification of WT and *pac* ^−/−^R donor cells in WT hosts (*n* = 5 embryos in each group, numbered 1–5 in graph) at 0 h (∼48–51 hpf) and termination of migration (40 h later) does not show decreased GC numbers, indicating that cell death occurs after migration during terminal GC differentiation. cb, cerebellum; ot, optic tectum.

Interestingly, at 6 dpf the GFP-expressing granule neuron population was largely diminished in gata1:GFP/*pac* ^−/−^R cerebella (Figure 12B) when compared to WT gata1:GFP larvae (Figure 12A, yellow arrowheads). Moreover, remaining gata1:GFP/*pac* ^−/−^R GCs lacked prominent parallel fibers and axonal projections to the crista cerebellaris (compare Figure 12A, 12B, white arrowheads) [25], indicating that not only terminal differentiation but also maintenance of granule neurons may be affected. We therefore quantified cell death in the cerebellum by acridine orange staining during different developmental stages. No difference could be detected between WT (0.83±0.9 dead cells, *n* = 12 embryos) and *pac* ^−/−^R embryos (0.92±0.9 dead cells, *n* = 12 embryos) at the immediate onset of GC migration at 48 hpf (unpublished data). Again this finding argues for a proper rescue of *pac* ^−/−^ mutant embryos, which normally show significantly increased levels of cell death at these developmental stages [7]. When major GC migration ceased (between 4 and 5 dpf), *pac* ^−/−^R embryos showed increasing levels of cell death (Figure 12D, 12E, *n* = 8 embryos), being five-fold higher in comparison to WT embryos (Figure 12C, 12E, *n* = 10 embryos). These findings imply that impaired terminal differentiation of cerebellar GCs and the lack of their maintenance in *pac* ^−/−^R embryos contribute to increased cell death. To address whether cell death already initiated in migrating GCs before final positions were reached, we counted the number of either WT or *pac* ^−/−^R GCs in transplantation embryos at migration onset and termination. Similar to the WT controls we did not observe a decrease in the numbers of *pac* ^−/−^R GCs during this migratory period of about 40 h (Figure 12F). These findings therefore suggest that death of Cadherin-2 deficient GCs occurs after migration during the period of granule neuron maturation.

## Discussion

The coordinated migration of neurons is essential for their correct positioning and proper integration into functional neuronal networks of the mature brain. We found that GC migration in the developing zebrafish cerebellum occurs across the dorsal surface in a homophilic manner by the formation of chain-like structures. The formation of such migratory chains may ensure a caudo-rostral migratory direction of co-migrating GCs emanating from the same region of the spatially patterned URL in zebrafish and prevent a lateral dispersion of GCs into aberrant cerebellar regions [25],[37],[38]. Glial cells do not contribute to migratory chains during the peak of this embryonic GC migration. Previous time-lapse studies in our lab have revealed that embryonic GC migration significantly diminishes at day 4 and 5 post fertilization with cerebellar circuits being established [25]. Nevertheless, during these periods and until adulthood GC migration continues in zebrafish at lower rates. GC migration in adult zebrafish has been proposed to occur in a radial glia-dependent manner [39],[40] and we have observed similar migration modes for GCs in juvenile fish (unpublished data). Thus, the homophilic chain-like GC migration in zebrafish embryos is likely replaced by a later heterophilic glia-dependent radial GC migration mode in juvenile zebrafish. In this paper we have addressed the embryonic migration of GCs from the URL whereas the migratory pathways of GCs during adulthood and their underlying cellular and molecular mechanisms remain to be resolved.

During neurulation, Cadherin-2 is required for the proper development of the URL like for other ventricular zones throughout the CNS [7],[15] by mediating polarization of neural progenitors. When the neurulation phenotype in *parachute* embryos was rescued, GCs remained polarized despite the later progressive lack of Cadherin-2 expression. These findings indicate that after neurulation, polarization of GCs is mediated in a Cadherin-2-independent manner. Recently, a second classic type I Cadherin, Cadherin-6, was found to be expressed in the differentiating zebrafish cerebellum, however its expression becomes confined to the URL at 48 hpf and is absent in migrating GCs [41]. Thus with respect to the URL, functional redundancy among Cadherin molecules could represent an alternative explanation for persistent polarization of Cadherin-2 depleted GCs in the URL. Our TEM observations showed, however, that Cadherin-2 deficient cerebellar cells apart from the URL lacked adherens junctions, indicating that Cadherin-2 is the predominant type I classic Cadherin expressed in zebrafish GCs during migration and differentiation. During these processes we found that Cadherin-2 directly regulates the coherence and directionality of GC migration by mediating stable cell-cell contacts for the formation of homophilic chain-like structures and by maintaining cellular polarity of GCs.

Transplantation experiments indicated that Cadherin-2 acts autonomously with respect to GC migration in chain-like structures and regulates directionality of cohesively migrating GCs. Migration of Cadherin-2 deficient GCs occurred in a non-cohesive and non-directional manner when transplanted into a WT cerebellar environment, whereas WT GCs migrated properly in chain-like structures in a Cadherin-2 deficient host cerebellum. Although only small numbers of cells were transplanted to host embryos we cannot fully exclude that donor cells also contributed to other cerebellar structures and provide an additional non-cell autonomous function of Cadherin-2 in regulating GC migration.

A detailed study of the subcellular dynamics of zebrafish GC migration has not been performed so far, but many of our observations from high-resolution time-lapse imaging, such as saltatory nuclear movements into the leading edge and trailing of the cell soma (M. Distel & R. W. Köster, unpublished data), are in good agreement with a nucleokinetic migration mode of zebrafish GCs. In homophilically migrating neurons nucleokinesis is largely mediated by the microtubule cytoskeleton, which tethers nucleus and centrosome (the MTOC) to adhesion sites in the plasma membrane [20],[21]. It has been thought that the disassembly of adherens junctions by neuronal cells prior to migration is required to reduce cell-cell interactions and to allow for flexible modulations of cell shape thereby enabling cellular motility. However, maintenance of adherens junctions is also required for cohesive cell behavior and cell polarity [30]. Thus, Cadherin-2 mediated functions may be regulated by dynamic relocation inside migrating GCs. This can occur via vesicle transport [33],[42], flow along reorganizing actin filaments [43],[44], or endocytosis and redeployment [45]. Using a fluorescent Cadherin-2 reporter we showed that GC elongation prior to forward migration is accompanied by a shift of this Cadherin-2 reporter to the front compartment of the cell. In addition, direct tracing studies of this fluorescent Cadherin-2 reporter in migrating GCs suggest that Cadherin-2 mediated adhesion is relocated along the cytoplasmic membrane toward the leading edge during GC forward migration as the steady levels of fluorescent emission indicate a collective movement of Cadherin-2 molecules as stable clusters. It has to be noted though that this reporter may not entirely reflect the endogenous expression of Cadherin-2 and the data have to be interpreted cautiously. Such time-resolved in vivo quantification experiments will have to await homologous recombination experiments, replacing endogenous Cadherin-2 with a fusion protein of Cadherin-2 and a fluorescent reporter. The slow but steady forward movement of clusters of the Cadherin-2 reporter is in good agreement with the slow migratory velocity of zebrafish GCs with one forward step occurring in about 45 to 60 min (Figure 10A–10F) [27]. The accumulation of Cadherin-2 in the anterior compartment of the GC could thus mediate a microtubule-mediated stabilization of the centrosome in front of the nucleus toward the leading edge. This in turn stabilizes the chosen direction via reorganization of the microtubule system [46], thereby maintaining the directionality of GCs during nucleokinetic cell migration. Lack of Cadherin-2 and consequently adherens junctions will therefore lead to an impaired anchoring and randomized positioning of the centrosome in zebrafish GCs (Figure 8D, 8F), thereby resulting in directionality defects as observed in our studies. Our findings may therefore offer an explanation of how nucleokinetic movements are stably maintained in a specific direction during neuronal migration. Intriguingly, recent in vitro studies of migrating rat astrocytes and endothelial cells on Cadherin-2 coated patterned surfaces showed that asymmetric Cadherin-2 localization induces centrosome relocalization and importantly nuclear movements in the direction of cell-cell contacts, strongly supporting our observations. [47]. In addition, recent studies demonstrated that tangential migration of cerebellar GCs in mouse is regulated by the transmembrane ligand Semaphorin 6A and its receptor Plexin-A2 [48]. In an elegant study, this signaling cascade was demonstrated to affect centrosome movements and its stable positioning in front of the nucleus during tangential nucleokinetic migration [22]. Interestingly, Cdk5 is a downstream target of Plexin-A2 signaling and this kinase has been shown to modulate Cadherin-2 adhesion and could therefore promote neuronal migration by stimulating the turnover of adherens junctions [49]. It will thus be interesting to investigate whether Plexin-signaling and Cadherin-2 mediated adhesion are linked in migrating zebrafish GCs.

Improper GC positioning via long-distance migration can result in compromised function and survival at later stages. For example, in several mouse mutants, including *weaver* mice, the mispositioning and ectopic differentiation of GCs is followed by their subsequent death [50]–[52]. This is in good agreement with our observations of a significantly increased cell death in cerebella with mispositioned Cadherin-2 deficient GCs at late stages of terminal GC differentiation. Currently, we cannot exclude that metabolic factors, such as insufficient oxygen or nutrient supply in *pac* ^−/−^R embryos, contribute to or cause this increase in cell death. However this seems unlikely, because blood vessels are functional in *pac* ^−/−^R embryos, as revealed by quantum dot microangiography (unpublished data) [29]. Furthermore, blood supply becomes essential for zebrafish larvae only after 7 dpf [53],[54]. In contrast, we observed a significant increase in cell death and loss of zebrafish gata1:GFP expressing GCs in *pac* ^−/−^R larvae at about 5 dpf, while *pac* ^−/−^R larvae survive at least until 7 dpf. Thus the improper positioning of GCs in the Cadherin-2 deficient cerebellum could lead to their impaired terminal differentiation followed by compromised cerebellar integrity, function, and subsequent cell death. Alternatively, independent of migration Cadherin-2 is required in GCs at later stages of terminal differentiation. For example, Cadherin-2 has been shown to play an important role as a transsynaptic adhesion molecule during synaptogenesis [33],[55],[56]. Such a later essential function of Cadherin-2 for zebrafish GC survival is supported by our findings that during or just after migration Cadherin-2 deficient GCs do not show signs of increased cell death. Furthermore, impaired migration and ectopic positioning of granule neurons in several mouse mutants does not affect GC survival [22],[57]. Also an indirect cause of GC death—for example due to the improper differentiation of Cadherin-2 depleted Purkinje cells—cannot be excluded currently. Like in other organisms, it is technically challenging in zebrafish to manipulate Cadherin-2 function exclusively during GC maturation, which would allow discriminating between the distinct stages of migration and synaptogenesis. Such an analysis has to await GC-lineage specific and temporally controlled ablation of Cadherin-2 function. With the recent advances in enhancer trapping and combinatorial Gal4-genetics in zebrafish, such a sophisticated study may soon become available [58],[59].

A key question will be to identify the mechanisms and molecules that guide migrating zebrafish GCs toward the MHB. Early during zebrafish cerebellar development (24 hpf), when the width of the cerebellar primordium covers about two to three cell diameters, neural progenitors span the entire cerebellum from the URL to the MHB [27]. With ongoing cerebellar growth and differentiation, this initial orientation of individual neural progenitors could be inherited to chain-like structures by first forming chains of two progenitors, which span the cerebellum together. Subsequently more and more GCs are added to the chains as cerebellar growth and differentiation progresses. Although this model could explain how GCs obtain their initial directionality, this mechanism is error-prone as proper migration is dependent on the integrity of the migratory chain-like structures at all times.

More attractive is the finding that the Slit-receptor Roundabout upon binding to its ligand was shown to interact with Cadherin-2. The ligand-receptor complex then inhibits Cadherin-2 adhesive properties by removing b-Catenin from the adhesion complex [60]. Moreover, Slit-2 signaling was recently shown to mediate migratory turns in explanted GCs away from the ligand source, likely involving directional changes in nucleokinesis [61]. Members of the Slit-ligands and Robo-receptors are expressed in the differentiating zebrafish cerebellum during GC migration [62], with *slit1a* being expressed in the URL [63]. Thus, Slit-signaling in the zebrafish URL could weaken Cadherin-mediated adhesion in the trailing edge of GCs and drive their migration along chain-like structures towards the MHB, thereby steering from the rear.

Alternatively, instead of guidance mechanisms affecting individual cells, GC migration could be driven by collective cell behavior, acting on migratory GC chains. Here cells within a chain might respond to guidance cues as a group, thus requiring the integrity of the chain by Cadherin-2 mediated adhesion for a proper directional migratory response. Such collective migration behavior for example was observed for border cells in *Drosophila* egg chambers or migrating cells in the lateral line primordium of zebrafish embryos [64],[65]. These questions will have to be answered by future in vivo studies on the cell biology of GC migration.

## Materials and Methods

### Maintenance of Fish

Raising, spawning, and maintaining of zebrafish lines was performed as described previously [24]. For simplicity, stable transgenic gata1:GFP embryos (strain 781) [25] will be referred to as gata1:GFP; *pac^R2.10^* as *pac* ^−/−^; and rescued *pac* ^−*/*−^ mutant embryos [7] as *pac* ^−/−*-*^R throughout the paper.

### Plasmid Construction and RT-PCR

Expression constructs were cloned into pBluescriptII (Stratagene) or pCS2+ [66] vectors.

pCS-Cdh2 was kindly provided by Matthias Hammerschmidt [7].

p14xUASE1b:Cdh2-EGFP was kindly provided by James Jontes [33].

p14xUASE1b:Cdh2-mCherry: p14xUASE1b:Cdh2-EGFP was digested AgeI/NotI to replace EGFP with an AgeI/NotI PCR-amplified fragment of mCherry [67]. The following primers were used: Cherry-up: ATA CCG GTC ATG GTG AGC AAG GGC and Cherry-low: GGG CGG CCG CTC TTA CTT GTA CAG C.

pCS-Cdh2ΔN-mCherry: p14xUASE1b:Cdh2ΔN-EGFP [33] was digested AgeI/NotI to replace EGFP with an AgeI/NotI PCR-amplified fragment of mCherry (see above). Subsequently, Cdh2ΔN-mCherry was cloned as a blunted Asp718/NotI-fragment into pCS2+ [66], which had been digested with StuI/SnaBI.

pCS-Cdh2ΔC-mCherry: To delete the cytoplasmic tail of Cadherin-2, a CdhΔC fragment was PCR-amplified from p14xUASE1b:Cdh2-EGFP [33] and cloned as BamHI/SalI-fragment in frame into a previously generated pCS2+-mCherry construct. The following primers were used: Cd-up: ACG GGA TCC CCA CCA TGT ACC CCT CCG GAG GCG TGA TGC T and Cd-low: TGT GTC GAC TTA TCC CGT CTC TTC ATC CAC ATC ACA AAC A.

pCS-Cdh2ΔEC2-4ΔC-mCherry: pCS-Cdh2ΔC-mCherry was digested with BglII and religated after Klenow-mediated 5′ fill-in.

pB8xHSE: pBSK+8xHSE:GFP [34] was digested with SalI to remove GFP, followed by vector religation.

pBTol8xHSE:mem-RFP: a membrane targeted variant of mRFP1pA containing a myristinylation site at its N-terminus was excised from a pCS2+ vector and cloned under control of the heat-shock elements in vector pB8xHSE, both of which had been digested ClaI/NotI. Subsequently the 8xHSE:mem-RFPpA expression cassette was transferred into the pBTolRG-vector [68].

pB8xHSE:Cdh2ΔEC2-4ΔC–mCherry: A Cdh2ΔEC2-4ΔC–mCherrypA-fragment was excised from pCS-Cdh2ΔEC2-4ΔC-mCherry by a BamHI/NotI-digest and cloned under control of the heat-shock elements in vector pB8xHSE, which had been digested with BglIII/NotI.

pCS-Cdh2ΔEC2-4ΔC-dEosFP: pCS-Cdh2ΔEC2-4ΔC-mCherry was digested SalI/XhoI to remove mCherry. A PCR-amplified SalI-fragment of dEosFP was cloned in frame behind Cdh2ΔEC2-4ΔC. For PCR, pcDAN3-Flag1 EosFP [35] and following primers were used: Eos-up: GCT GTC GAC CAT GGA CTA CAA AGA CGA TGA CGA TA, Eos-low: TTA GTC GAC ACT ATA GAA TAG GGC CCT TAT CGT CTG G.

pB8xHSE:Cdh2ΔEC2-4ΔC–dEosFP: A Cdh2ΔEC2-4ΔC–dEosFPpA-fragment was excised from pCS-Cdh2ΔEC2-4ΔC-dEosFP by a BamHI/Asp718(blunt)-digest and cloned under control of the heat-shock elements in vector pB8xHSE, which had been digested BglII/NotI(blunt).

pCS-GalTA: A cDNA-fragment containing the Gal4-DNA binding domain fused to a minimal VP16-transactivation domain was cloned into EcoRI/XhoI sites of pCS2+.

pCS-Centrin2-tdTomato: tdTomato was excised from pRSETtdTomato [67] and cloned into BamHI/EcoRI-sites of pCS2+. The open reading frame of zebrafish *centrin2* (acc. nr.: EU183505) was cloned by RT-PCR using total RNA from adult brain and the following primers: centrin-up: TTG GAT CCA TGG CGT CCG GCT TCA GGA A, centrin-low: TTT CTA GAT CAG TAC AGA TTG GTT TTC TTC. The fragment was subcloned into the pCRII-Topo-vector (Invitrogen, San Diego, CA) and sequenced. Subsequently, the Centrin2-fragment from pCRCentrin2 was cloned in frame in front of tdTomato as HindIII/BamHI fragment.

pBU-Centrin2-tdTomato: A blunted HindIII/EcoRI Centrin2-tdTomato-fragment was cloned behind a Gal4-binding UAS-consensus site into blunted EcoRI/SnaBI pB1xUASE1b-vector (M. Distel & R. W. Köster, unpublished).

Further details of cloning procedures are available on request.

### Expression Analysis and Fluorescent Labeling

ISH: ISH was performed on whole-mount embryos as described [25].

Immunohistochemistry: Mouse anti-BLBP (1∶1,500, kindly provided by Nathaniel Heintz) and rabbit anti-GFP (1∶500, Torrey Pines Biolabs) antibodies were detected with Cy-5 conjugated anti-mouse IgG and Cy-2-conjugated anti-rabbit IgG (1∶250, Dianova), respectively.

Fluorescent stains: Bodipy Ceramide Fl C5 and TOPRO (both Invitrogen) were dissolved in DMSO for membrane and nuclear staining, respectively. Embryos were fixed in 4% paraformaldehyde, dechorionated, and soaked overnight in 1 µM dye in PBS. To label dead cells, living WT and *pac* ^−*/*−^R embryos were soaked for 20 min in 5 µg/ml acridine orange solution (Invitrogen)/30% Danieau at 4 dpf and 5 dpf. Incubation of embryos in these different dyes was followed by three washes (1 min each) in 30% Danieau and images recorded using an inverted LSM510 Meta confocal microscope (Zeiss).

*DiI labeling*: DiI crystals (Invitrogen) were applied with a sharpened tungsten needle to neuroepithelial cells in the URL of 48 hpf embryos, embedded in 1.2% ultra-low melting agarose (Sigma-Aldrich). After recovery in 30% Danieau for 16 h at 28°C, the embryos were imaged using an inverted LSM510 Meta confocal microscope (Zeiss).

### TEM

TEM was performed as described in detail [69].

### Microinjection and *pac*R2.10 (*pac* ^−*/*−^) Rescue Experiments

Capped mRNA was synthesized from pCS2+ expression plasmids using the Message Machine Kit (Ambion). For mosaic membrane labeling, about 120 pg of lynGFP mRNA was injected into individual blastomeres of 8–16 cell stage embryos. For *pac* ^−*/*−^ rescue experiments, 70 pg of *cadherin-2* mRNA was injected into early 1-cell stage embryos. For other mRNA expressions in gata1:GFP GCs, 100 pg of messenger RNA encoding for membrane-bound RFP and Cdh2ΔEC2-4ΔCmCherry was injected into 1-cell stage embryos. For DNA injections, expression constructs were purified using GeneClean Turbo kit (Q-BIOgene) and 50–100 pg of plasmid DNA was injected into 1-cell stage embryos. For Gal4-mediated centrosome labeling, 15 pg of pCS-GalTA-activator and pBU-Centrin2- tdTomato-effector construct were co-injected into 1-cell stage embryos.

### Temperature-Induced Expression of Cadherin-2 Variants

Plasmids (100pg) encoding 8xHSE constructs were injected into 1-cell stage embryos. To activate transcription, 5 ml of 30% Danieau/PTU containing 50 hpf embryos were transferred into a 15 ml falcon tube and incubated at 39.7°C in a pre-heated water bath for 3×30 min. Each incubation time was followed by a recovery period for 30 min–1 h in the same solution. Finally, the embryos were transferred into fresh Danieau and maintained at 28°C for >2 h until protein expression was observed.

### Genotyping of *pac*R2.10 Embryos

Genotyping of *pac*R2.10 (*pac* ^−/−^ embryos by RT-PCR) was performed as described [7] using forward primer (−128 bp upstream of start-ATG) ATC AGT GCC AGA GAG AGA CGG AGG AA CGA and reverse primer (+91 bp within exon1) GCG GGA TTG GTT GTA CTC GTT CTC GGT GA.

### Western Blot Analysis

Embryonic protein extracts were prepared from ten pooled WT or *pac* ^−/−^ embryos, dissolved in 1xLaemmli buffer (Bio-Rad) and separated on a 10% acrylamide gel. The gel was blotted onto a PVDF membrane, which was probed with Cdh2 (1∶100 dilution, generated by Elizabeth Krämmer, Helmholtz Zentrum Muenchen) and rat anti-Tenascin (1∶1,000, kindly provided by Elizabeth Krämmer, Helmholtz Zentrum München). For detection, horse raddish peroxidase conjugated anti-mouse IgG (1∶500) and anti-rat IgG (1∶500) antibodies (both Dianova) were used, followed by ECL detection (BD Biosciences).

### Co-Immunoprecipitation

Protein extracts from Pac2 cells transfected with (a) p14xUASE1b:Cdh2-EGFP/pCSGalTA, (b) p14xUASE1b:Cdh2-mCherry/pCS-GalTA, or (c) pCS-Cdh2ΔEC2-4ΔCmCherry were prepared and 20 µg of total protein from each lysate analyzed by Western blotting. 500 µg protein, 1 µg mouse anti-GFP antibody (Invitrogen), and 10 µl Protein G Sepharose resin were incubated for 3 h at 4°C, washed 3 times in lysate buffer, and after denaturing in 0.1% SDS running buffer (2 min, 95°C) analyzed on a 10% acrylamide gel. For detection of bound protein, rabbit anti-mRFP1 (1∶2,000, Chemicon) and chicken anti-GFP (1∶2,000, Aves) antibodies were used.

### Genetic Mosaic Analyses

One-cell stage gata1:GFP donor embryos were injected with rhodamine-dextran 10,000 MW (Invitrogen). *Pac* ^−*/*−^ mutant donors were first injected with *cdh-2*-mRNA to rescue neurulation defects. For transplantations, embryos were dechorionated at the 4- to 16-cell stage using Pronase (Roche) and placed into agarose molds, covered with 30% Danieau or Ringers. For transplantation, a small group of cells (5–20) at sphere-stage (4 hpf) was transferred into the animal pole of host embryos using pulled and polished 1 mm outer diameter (OD) transplantation needles without filament. For identification of mutant hosts or donors, embryo pairs were raised together and screened at 24 hpf to identify their genotype using RT-PCR. Transplantation efficiencies were monitored in assessing the incorporation of rhodamine-labeled cells into host embryos.

### Electroporation

For single-cell electroporation, 48 hpf embryos were embedded in 1.2% ultra-low melting agarose and covered with 30% Danieau/PTU. To prepare the electroporation electrode, a silver wire microelectrode (World Precision Instruments) was inserted into a pulled 1 mm OD glass capillary that was filled with 100 ng/µl of purified plasmid DNA, containing 0.01% Fast Green (Sigma). The capillary electrode was positioned onto a single cell in the URL. A platinum wire was placed on the opposite site in direction of the desired DNA transfer, and two trains of square pulses (255 V, 600 µs separated by 100 µs) were delivered with an ECM810 electroporator (BTX Harvard Apparatus). Embryos were allowed to recover until protein expression was observed (6–8 h).

### In Vivo Time-Lapse Imaging

For time-lapse imaging, WT, *pac* ^−*/*−^ and *pac* ^−/−^R gata1:GFP embryos were mounted in 1.2% ultra-low melting agarose (Sigma-Aldrich) in glass-bottom Petri dishes and covered with 30% Danieau/0.15 mM PTU/0.01% Tricaine as anesthetic. Image recording was performed using an inverted LSM510 confocal microscope (Zeiss) and C-Apochromat water-immersion objectives (40x, 63x) as described [70]. Videos were assembled using QuickTime Player 7.2Pro. For quantifications of GC migration behavior, digital images were processed using Adobe Photoshop 7.0D and the NIH open source software IMageJ1.34s [71]. Cell tracings were performed using the ImageJ Manual Tracking plug-in.

### Quantifications and Statistical Analyses

For quantification of time-lapse results, cells were randomly chosen based on the ability to sufficiently trace their migratory pathway. All statistical analyses were performed with the GraphPad Prism 4 software.

Contact stabilities were determined by measuring the contact duration between a GC pair in gata1:GFP WT and *pac* ^−/−^R embryos over 120 min. For statistical analysis, the unpaired two-tailed Student *t* test was used (*n* = 29 analyzed GC pairs, 5 embryos in each group).

LWRs present the ratio of the longest dimension of an elongated GC (not including the leading edge), divided by the widest distance between the two lateral edges. One-way ANOVA and multiple comparisons post-test were used for WT (*n* = 29), *pac* ^−/−^R (*n* = 34), *pac* ^−/−^ (*n* = 26), and non-migrating terminally differentiated GC comparisons (*n* = 34).

Migration distance and velocity: Linear distances were determined by calculating the shortest distance between the initial position of a GC (e.g., 50 hpf) and its end position after 4 h (e.g., 54 hpf). Total migration distances were determined by tracing paths of individual GCs over 4 h using the ImageJ manual tracking tool. Velocities are represented in distance (µm) per minute. Statistical analyses were performed using the two-tailed Student's *t* test.

Centrosome positions were determined using two parameters: (1) CS position relative to cerebellar boundaries and (2) CS position relative to cell morphology. (1) To quantify whether the centrosome was predominantly located in direction of GC migration, cells were divided into four quadrants related to MHB, URL, and medial/lateral cerebellum (example shown in Figure 8D). (2) To quantify whether the centrosome preferentially located to a certain cell compartment during GC migration, each cell was divided into four quadrants relative to cellular morphology (LE, rear, left/right lateral membrane) (example is shown in Figure 8F).

Leading edge–centrosome relationship: The relationship between leading edge and centrosome in migrating GCs was analyzed over 90 min by time-lapse analysis; positive values represent forward movements and negative values rearward movements. From time point to time point, movements of the leading edge and centrosome were characterized as either occurring in the same or opposite direction and plotted for WT and *pac* ^−/−^R GCs, respectively.

Fluorescence intensities in GCs expressing Cdh2ΔEC2-4ΔCmCherry were quantified by dividing each GC into three equal portions (front, medial, and rear compartment), based on their total calculated area (Zeiss LSM software). The total fluorescence intensity was determined and a percentage for each compartment calculated. Represented are the means of five cells, derived from five embryos. One-way ANOVA was used for statistical analysis.

## Supporting Information

Figure S1 **Temporal rescue of** ***pac*** **^−/−^ embryos is restricted to developmental stages prior to cerebellar GC migration.** (A–I) Dorsal views of zebrafish embryonic heads at 24 hpf. (A–C) Light microscopy of cerebellar primordium in WT (A) and *pac* ^−/−^ (B) embryos that can be rescued by Cdh2-mRNA injection in *pac* ^−/−^R-embryos (C). (D–I) This rescue is confirmed by ISH showing the reconstitution of wnt1 dorsal midline expression (D–F) and *atonal1a *expression throughout the rhombic lip (G–I) in *pac* ^−/−^R embryos. (L) RT-PCR confirms *pac* ^−/−^ genotype in rescued mutant embryos (tail clip RT-PCR lanes 3+4 was performed on same embryos displayed in F and I, respectively). (J, K) Whole-mount ISH analysis of *cadherin-2* expression reveals that *cadherin-2* mRNA is hardly detectable in both *pac* ^−/−^ (J) and *pac* ^−/−^R-embryos (K) at 24 hpf (dorsal views of hindbrain). (M, N) In contrast, Western blot analysis of total embryo extracts including the membrane fractions detects Cadherin-2 protein in *pac* ^−/−^R-embryos at 24 hpf, with levels comparable to WT embryos (lane 1). By 48 hpf, at the onset of GC migration, Cadherin-2 protein is mostly degraded in *pac* ^−/−^R-embryos (M, lane 6, black arrow) and completely absent at 72 hpf (N, lane 6). While Cdh2 protein derived from mRNA injections is mostly degraded at 48 hpf, Cdh-2 protein is continuously expressed from plasmid DNA (M, lane 7). Loading controls: TenascinR in (M) and β-Tubulin in (N). cb, cerebellum; mes, mesencephalon; rh, rhombencephalon.
(5.98 MB TIF)

Figure S2 **Cloning and verification of a Cadherin-2 variant as in vivo adhesion reporter protein.** (A) Schematic representation of different Cadherin-2 variants (black diamond in EC1 represents cis-dimerizing activity). (B, C) Full-length Cadherin-2 (B) and dominant-negative Cdh2ΔN (C) mRNA-injected embryos analyzed by ISH for *atoh1a* expression at 24 hpf show severe morphological defects in the hindbrain (dorsal view, anterior is left). (D–F) In contrast, embryos injected with Cdh2ΔEC2-4ΔC:mCherry (D, lateral view of head, overlay with mCherry expression) do not reveal defects, neither by morphology nor by expression of *wnt1* (E) or *atoh1a* (F). Furthermore, mRNA-injection of this variant was unable to rescue *pac* ^−/−^R embryos (unpublished data). (G–I) Single optical section (1 µm) of a cell in the URL at 65 hpf, co-electroporated with full-length Cdh2:GFP and Cdh2ΔEC2-4ΔC:mCherry plasmid DNA. (G) Full-length Cadherin-2 preferentially clusters in the anterior cell and along the lateral plasma membrane (white arrowheads). The Cdh2ΔEC2-4ΔC:mCherry reporter variant (I) colocalizes with full-length Cadherin-2:GFP in the same regions (see GFP and mCherry overlayed in H). (J) Co-immunoprecipitation (IP) using the Cadherin-2:GFP fusion protein as bait (Input: lane 2) reveals direct interaction between Cdh2ΔEC2-4ΔC:mCherry and Cadherin-2:GFP (IP: lane 2). Full-length Cadherin-2:mCherry used as positive control shows similar interactions (IP: lane 4). (K–L) Bodipy Ceramide membrane staining (K) overlayed with Cdh2ΔEC2-4ΔC:mCherry fluorescence (L) expressed from injected mRNA shows membrane localization of this variant (L, M) and intact cellular morphologies in the cerebellum at 48 hpf. LRL, lower rhombic lip; MHB, mid-hindbrain boundary; URL, upper rhombic lip.
(2.73 MB TIF)

Video S1 **This movie shows the homophilic migration of zebrafish cerebellar GCs.** First part: Colored dots depict migrating GCs in individual chains. Arrows point to single GCs within each chain that adhere to other granule cells as they move along each other toward the MHB. Second part: shows pseudo-colored GCs during chain migration.
(0.14 MB MOV)

Video S2 **This movie shows a dorsal view of the antero-lateral migration pathway of cerebellar GCs in a zebrafish gata1:GFP transgenic embryo.** GCs migrate from the URL toward and along the MHB.
(0.35 MB MOV)

Video S3 **This movie shows a dorsal view of migrating GCs in gata1:GFP/** ***pac*** **^−/−^ embryos.** The lack of Cadherin-2 results in impaired migration of cerebellar GCs. Most GCs, although motile, remain close to the URL and eventually form an ectopic cluster in the dorsal cerebellum.
(0.46 MB MOV)

Video S4 **This movie shows the cohesive and directional migration of WT gata1:GFP GCs.** Migrating GCs were recorded in one cerebellar lobe. The migratory routes of individual GCs were traced using ImagJ1.34s software (Manual Tracking Tool plug-in) and appear in the second part of the movie. Arrows in the final image depict the overall migration direction of traced GCs, demonstrating their highly coherent and directional migration behavior.
(0.36 MB MOV)

Video S5 **This movie shows the loss of cohesion and directional migration of GCs in** ***pac*** **^−/−^R embryos.** The migratory routes of individual gata1:GFP/*pac* ^−/−^R GCs were traced using ImagJ1.34s software (Manual Tracking Tool plug-in) and are shown in the second part of the movie. The final arrows depict the overall migration direction of traced GCs, demonstrating that lack of Cadherin-2 in rescued *pac* ^−/−^R embryos leads to a loss of directional and cohesive migration.
(0.20 MB MOV)

Video S6 **WT gata1:GFP GCs co-expressing membrane targeted RFP.** This movie shows that GFP expression in transgenic gata1:GFP GCs reliably outlines cell morphologies, similar to membrane-localized RFP fluorescence (see white arrows). RL, cerebellar rhombic lip.
(0.62 MB MOV)

Video S7 **GC migration of WT gata1:GFP donor cells after transplantation into WT host embryos (one cerebellar lobe is shown for simplicity) shows that donor-derived GCs follow antero-lateral routes from the URL toward the MHB of the host embryo.** This is confirmed by tracing of individual donor cells.
(0.63 MB MOV)

Video S8 **Migration of gata1:GFP/** ***pac*** **^−/−^R GCs after transplantation into WT host embryos (one cerebellar lobe is shown for simplicity).** Migrating gata1:GFP/*pac* ^−/−^R GCs fail to migrate in a directional manner as confirmed by tracing individual mutant donor cells. These tracks reveal the loss of cohesive and directional migration of Cadherin-2 deficient GCs in a WT environment.
(0.80 MB MOV)

Video S9 **Migration of WT gata1:GFP donor GCs in the cerebellum of a** ***pac*** **^−/−^R hosts, beginning at 50 hpf (one cerebellar lobe is shown for simplicity).** Donor-derived GCs migrate in chain-like structures (arrows) toward the MHB and maintain directionality, although surrounded by Cdh2-deficient cells.
(1.40 MB MOV)

Video S10 **This movie shows the impaired emigration of URL cells expressing a heat shock-induced dominant-negative Cadherin-2-mCherry variant (8xHSE:Cdh2**Δ**N-mCherry).** Red fluorescent URL-cells fail to join neighboring GC chains on their migratory routes toward the MHB. Only one cerebellar half is presented.
(0.10 MB MOV)

Video S11 **This movie shows that migration of GCs expressing a non-functional Cadherin-2-mCherry variant (8xHSE:Cdh2Δ2-4ΔC-mCherry) is similar to WT GC migration.**
(0.05 MB MOV)

Video S12 **This movie displays centrosome (CS) and leading edge (LE) dynamics of WT gata1:GFP GCs during migration.** Centrosomes were fluorescently labeled by co-expression of pCS-GalTA and pBU-Centrin2:tdTomato constructs in gata1:GFP GCs.
(0.21 MB MOV)

Video S13 **This movie displays centrosome (CS) and leading edge (LE) dynamics of Cadherin-2 deficient** ***pac*** **^−/−^R gata1:GFP GCs during migration.** Centrosomes were fluorescently labeled by co-expression of pCS-GalTA and pBU-Centrin2:tdTomato constructs in *pac* ^−/−^R gata1:GFP GCs.
(0.13 MB MOV)

Video S14 **This movie shows the dynamics of Cadherin-2 clusters in migrating GCs visualized by the expression of a Cadherin-2 reporter molecule (8xHSE:Cdh2Δ2-4ΔC-mCherry).** During elongation, Cadherin-2 clusters (white arrowhead) accumulate in the leading edge, while they diminish from the rear compartment (yellow arrowhead). As the GC moves forward Cadherin-2 clusters concentrate along the lateral cell wall in close apposition to the neighboring GFP-expressing GC. Eventually during a resting phase, Cadherin-2 clusters can be found in the rear compartment again (overlay of GFP is shown in the second part of the movie).
(0.16 MB MOV)

Video S15 **Cadherin-2 transport in the cytoplasmic membrane of migrating GCs.** This movie shows the directed forward transport of UV-converted Cdh2Δ2-4ΔC-EosFP along the cytoplasmic membrane of a cerebellar GC in a gata1:GFP embryo. Individual Cdh2Δ2-4ΔC-EosFP-clusters (white arrowhead) pass a neurite (yellow arrowhead) by translocating inside the cytoplasmic membrane toward the front of the GC. Note the forming trailing edge of the GC indicates initiation of forward movement.
(0.03 MB MOV)

## Abbreviations

AJ: adherens junction
dpf: days post fertilization
BLBP: Brain Lipid-Binding Protein
CNS: central nervous system
DLV: dorsal longitudinal vein
GFP: Green Fluorescent Protein
GC: granule cell
hpf: hours post fertilization
ISH: in situ hybridization
LWR: length-width ratio
MHB: midbrain-hindbrain boundary
MTOC: microtubule organizing center
OD: outer diameter
TEM: Transmission electron microscopy
URL: upper rhombic lip
WT: wild type
