# Supplementary figures and images for: Cadherin-2 Controls Directional Chain Migration of Cerebellar Granule Neurons
Source: PLoS Biol. 2009 Nov 10;7(11):e1000240. doi: 10.1371/journal.pbio.1000240 (PMC2766073; doi:10.1371/journal.pbio.1000240)

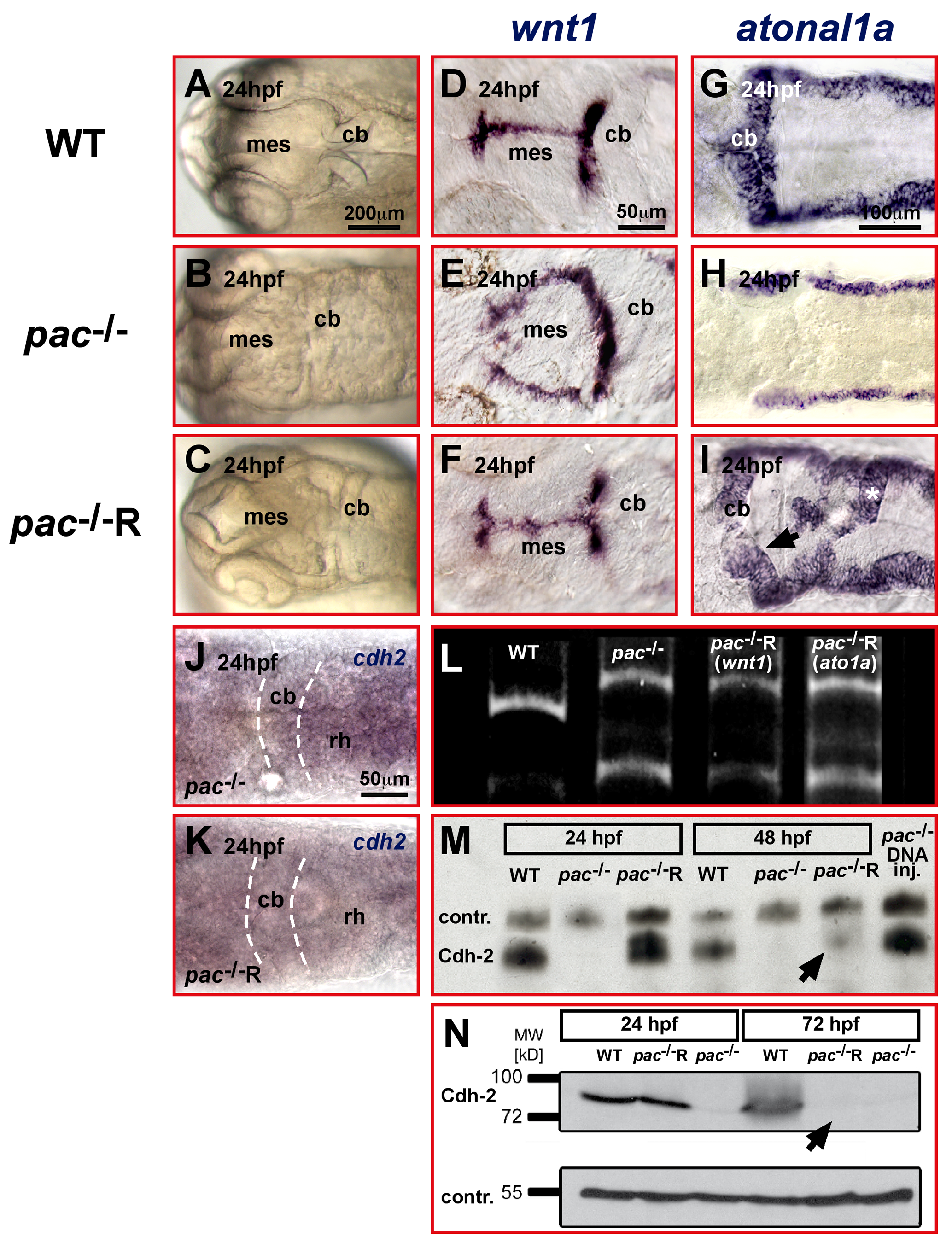

Supplement: Figure S1 — Temporal rescue of pac −/− embryos is restricted to developmental stages prior to cerebellar GC migration. (A–I) Dorsal views of zebrafish embryonic heads at 24 hpf. (A–C) Light microscopy of cerebellar primordium in WT (A) and pac −/− (B) embryos that can be rescued by Cdh2-mRNA injection in pac −/−R-embryos (C). (D–I) This rescue is confirmed by ISH showing the reconstitution of wnt1 dorsal midline expression (D–F) and atonal1a expression throughout the rhombic lip (G–I) in pac −/−R embryos. (L) RT-PCR confirms pac −/− genotype in rescued mutant embryos (tail clip RT-PCR lanes 3+4 was performed on same embryos displayed in F and I, respectively). (J, K) Whole-mount ISH analysis of cadherin-2 expression reveals that cadherin-2 mRNA is hardly detectable in both pac −/− (J) and pac −/−R-embryos (K) at 24 hpf (dorsal views of hindbrain). (M, N) In contrast, Western blot analysis of total embryo extracts including the membrane fractions detects Cadherin-2 protein in pac −/−R-embryos at 24 hpf, with levels comparable to WT embryos (lane 1). By 48 hpf, at the onset of GC migration, Cadherin-2 protein is mostly degraded in pac −/−R-embryos (M, lane 6, black arrow) and completely absent at 72 hpf (N, lane 6). While Cdh2 protein derived from mRNA injections is mostly degraded at 48 hpf, Cdh-2 protein is continuously expressed from plasmid DNA (M, lane 7). Loading controls: TenascinR in (M) and β-Tubulin in (N). cb, cerebellum; mes, mesencephalon; rh, rhombencephalon. (5.98 MB TIF) [file pbio.1000240.s001.tif]

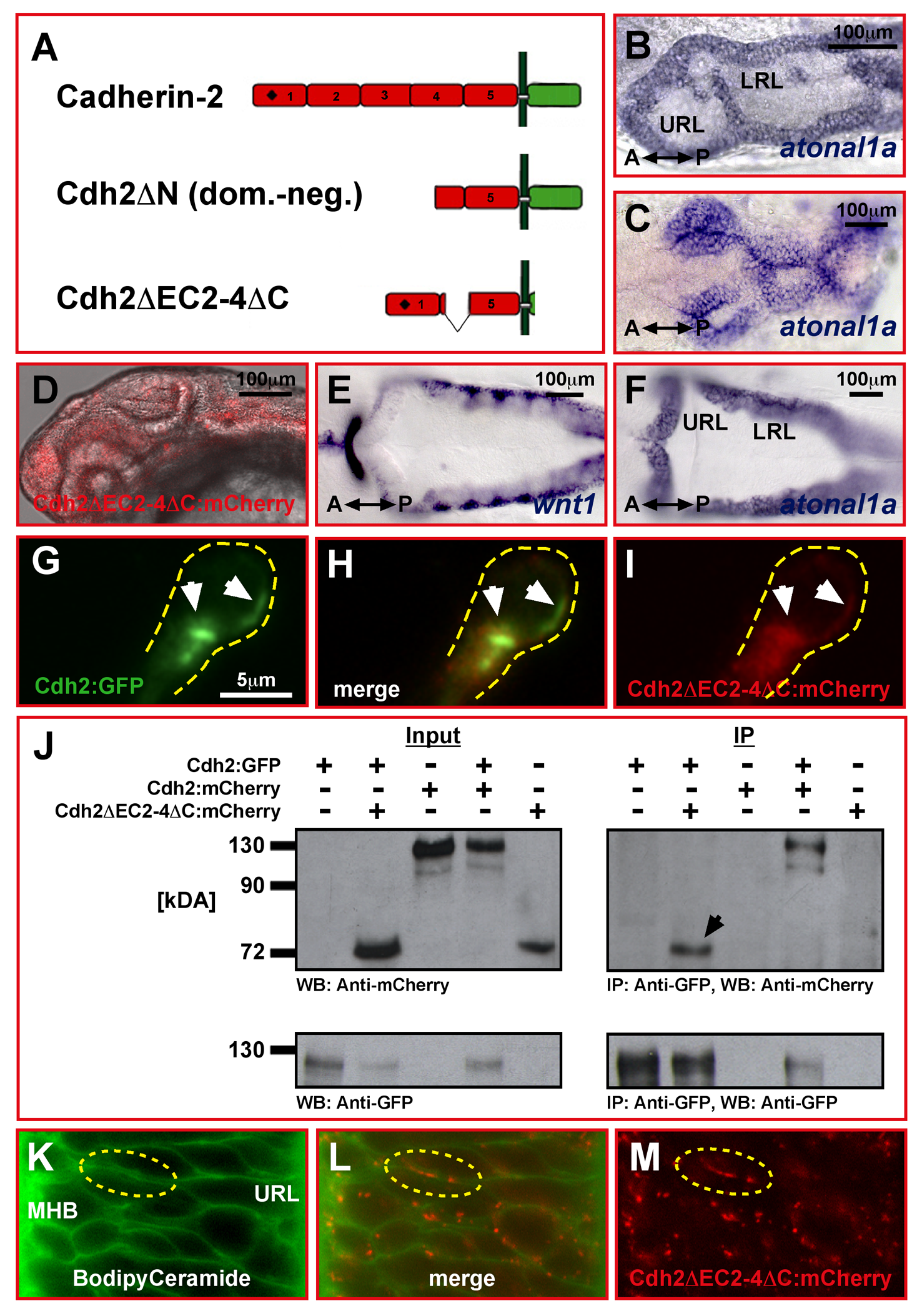

Supplement: Figure S2 — Cloning and verification of a Cadherin-2 variant as in vivo adhesion reporter protein. (A) Schematic representation of different Cadherin-2 variants (black diamond in EC1 represents cis-dimerizing activity). (B, C) Full-length Cadherin-2 (B) and dominant-negative Cdh2ΔN (C) mRNA-injected embryos analyzed by ISH for atoh1a expression at 24 hpf show severe morphological defects in the hindbrain (dorsal view, anterior is left). (D–F) In contrast, embryos injected with Cdh2ΔEC2-4ΔC:mCherry (D, lateral view of head, overlay with mCherry expression) do not reveal defects, neither by morphology nor by expression of wnt1 (E) or atoh1a (F). Furthermore, mRNA-injection of this variant was unable to rescue pac −/−R embryos (unpublished data). (G–I) Single optical section (1 µm) of a cell in the URL at 65 hpf, co-electroporated with full-length Cdh2:GFP and Cdh2ΔEC2-4ΔC:mCherry plasmid DNA. (G) Full-length Cadherin-2 preferentially clusters in the anterior cell and along the lateral plasma membrane (white arrowheads). The Cdh2ΔEC2-4ΔC:mCherry reporter variant (I) colocalizes with full-length Cadherin-2:GFP in the same regions (see GFP and mCherry overlayed in H). (J) Co-immunoprecipitation (IP) using the Cadherin-2:GFP fusion protein as bait (Input: lane 2) reveals direct interaction between Cdh2ΔEC2-4ΔC:mCherry and Cadherin-2:GFP (IP: lane 2). Full-length Cadherin-2:mCherry used as positive control shows similar interactions (IP: lane 4). (K–L) Bodipy Ceramide membrane staining (K) overlayed with Cdh2ΔEC2-4ΔC:mCherry fluorescence (L) expressed from injected mRNA shows membrane localization of this variant (L, M) and intact cellular morphologies in the cerebellum at 48 hpf. LRL, lower rhombic lip; MHB, mid-hindbrain boundary; URL, upper rhombic lip. (2.73 MB TIF) [file pbio.1000240.s002.tif]
